# Novel stem cell technologies are powerful tools to understand the impact of human factors on *Plasmodium falciparum* malaria

**DOI:** 10.3389/fcimb.2023.1287355

**Published:** 2023-12-19

**Authors:** Alena Pance, Bee L. Ng, Kioko Mwikali, Manousos Koutsourakis, Chukwuma Agu, Foad J. Rouhani, Ruddy Montandon, Frances Law, Hannes Ponstingl, Julian C. Rayner

**Affiliations:** ^1^ Wellcome Sanger Institute, Cambridge, United Kingdom; ^2^ School of Life and Medical Sciences, University of Hertfordshire, Hatfield, United Kingdom; ^3^ Bioscience Department, KEMRI-Wellcome Trust Research Programme, Kilifi, Kenya; ^4^ Wellcome Centre of Human Genetics, University of Oxford, Oxford, United Kingdom; ^5^ Cambridge Institute for Medical Research, University of Cambridge, Cambridge, United Kingdom

**Keywords:** stem cells, erythropoietic differentiation, malaria, *Plasmodium falciparum*, invasion, genome editing, reprogramming, haemoglobinopathies

## Abstract

*Plasmodium falciparum* parasites have a complex life cycle, but the most clinically relevant stage of the disease is the invasion of erythrocytes and the proliferation of the parasite in the blood. The influence of human genetic traits on malaria has been known for a long time, however understanding the role of the proteins involved is hampered by the anuclear nature of erythrocytes that makes them inaccessible to genetic tools. Here we overcome this limitation using stem cells to generate erythroid cells with an *in-vitro* differentiation protocol and assess parasite invasion with an adaptation of flow cytometry to detect parasite hemozoin. We combine this strategy with reprogramming of patient cells to Induced Pluripotent Stem Cells and genome editing to understand the role of key genes and human traits in malaria infection. We show that deletion of basigin ablates invasion while deletion of ATP2B4 has a minor effect and that erythroid cells from reprogrammed patient-derived HbBart α-thalassemia samples poorly support infection. The possibility to obtain patient-secific and genetically modifed erythoid cells offers an unparalleled opportunity to study the role of human genes and polymorphisms in malaria allowing preservation of the genomic background to demonstrate their function and understand their mechanisms.

## Introduction

Malaria is an infectious disease caused by several species of *Plasmodium* parasites that are transferred between humans by female Anopheline mosquitoes. The parasite life cycle is complex and involves multiple organs in host and vector, with a wide range of interactions between parasite and host at each step. Nevertheless, in the human host it is the asexual blood stages of the parasite that invade, develop and replicate in erythrocytes, causing a variety of pathological processes associated with malaria infection. The completion of reference genomes (Aurrecoechea et al., 2009), the development of genome editing technologies (Lo et al., 2013; Ran et al., 2013) and their adaptation to the parasite (Straimer et al., 2012; Ghorbal et al., 2014) have revolutionised our understanding of the parasite side of the blood cycle. These advances have enabled the identification of many parasite proteins involved in invasion (Cowman et al., 2017), as well as the formation of the parasitophorous vacuole, remodelling of the erythrocyte (Cowman et al., 2012; Weiss et al., 2015), and a much broader understanding of parasite blood-stage biology. On the host side, genome-wide association studies (GWAS) have identified multiple human genetic variants associated with differences in the severity of disease caused particularly by *Plasmodium falciparum*, the most virulent species affecting humans (Damena et al., 2019; Malaria Genomic Epidemiology N, 2019). These include many genes implicated in erythrocyte structure and function, such as the membrane protein Band 3, the red blood cell enzyme Glucose-6-Phosphate Dehydrogenase and the Haemoglobins, amongst others (Kariuki and Williams, 2020). However, despite population studies providing compelling evidence for protective effects of multiple human protein variants, the molecular mechanisms of these effects are frequently either undeciphered or disputed. This is mainly due to the limitations in accessing primary cell samples and the technical challenges of reproducing variants *in-vitro* in order to perform tightly controlled cellular studies.

There are two major hurdles for identifying host proteins that interact with malaria parasites and understanding their function. Firstly, erythrocytes are non-proliferative, terminally differentiated cells with a limited life span, which makes their long-term culture impossible. Thus, research has almost exclusively relied on clinical samples, with inherent difficulties of donor availability and variability as well as the impact of storage and transport on sample quality which impose limitations on the ability to perform detailed cellular studies. Secondly and perhaps most significantly for mechanistic studies, mature erythrocytes are anucleated and therefore gene editing technologies cannot be applied. Attempts to overcome these limitations have been developed in recent years using a variety of stem cell technologies. siRNA knock-down techniques in Haematopoietic Stem Cells (HSCs) have been used to study the specific role of Glycophorin A (GYPA) (Bei et al., 2010) and Basigin (BSG) (Crosnier et al., 2011) as well as to screen more broadly for erythrocyte proteins involved in *P. falciparum* growth and development (Egan et al., 2015). While a significant step forward, the applicability of this approach is curtailed by the restricted availability of HSCs, their limited proliferation capacity and the variable levels of knock-down that can be achieved. More recently, an immortalised adult erythroblast line able to proliferate and differentiate *in-vitro*, was established by transformation of erythroid progenitor cells with the human papilloma virus HPV16-dervied proteins HPV16-E6/E7 (Trakarnsanga et al., 2017). One such line (BEL-A) was combined with genome editing technologies to explore the mechanisms of Basigin involvement in *P. falciparum* invasion (Satchwell et al., 2019). This approach was also applied to peripheral blood samples and shown to generate cells permissive to *P. falciparum* and *P. vivax* invasion and amenable to genome editing studies (Scully et al., 2019). One potential disadvantage of this strategy is the viral transformation of the cells with its inherent genetic consequences that might be limiting on the long term, particularly when addressing natural genomic variation. Critically, studies using stem cell lines engineered to facilitate differentiation towards erythropoiesis do not offer the possibility of exploring specific genetic characteristics or complex genetic traits in their original genomic context, such as those found in patients or particular human populations.

Other approaches, such as the use of established Embryonic and induced Pluripotent Stem Cell lines (ESCs and iPSCs) that can be cultured (International Stem Cell et al., 2007) and genetically manipulated (Veres et al., 2014) while maintaining their pluripotency (Hendriks et al., 2020), could provide a versatile alternative with additional advantages. Stem cells have the potential to differentiate into any cell type, which would allow examination of the same genomic background on all parasite stages. Furthermore, the development of reprogramming techniques that revert terminally differentiated cells to pluripotency (Takahashi et al., 2007) makes it possible to generate iPSCs from any individual or sample. In this way, complex genotypes and rare variants, including non-viable mutations, can be brought into the lab and stored for unlimited studies. Genome editing to change or correct the mutations also becomes possible, offering a direct confirmation of their physiological role. Crucially, the use of stem cells also helps to overcome some of the ethical issues often encountered when pursuing molecular research in the malaria field.

In this work we developed a differentiation protocol to drive both ESCs and iPSCs towards erythropoiesis and produce cells that are competent for *P. falciparum* infection. This approach makes it possible to study a wide range of patient-derived cells simultaneously as well as make use of existing iPS lines, while also allowing the potential to incorporate genome editing of specific host genes and compare their effect on multiple different genomic backgrounds. Our protocol mimics more closely natural development by driving the pluripotent cells to mesoderm first, thus avoiding the commonly used embryoid body formation with the associated cell loss, and leading to a better yield of erythroid cells. To assess invasion of the *in-vitro*-derived cells, we established an assay to accurately quantify parasitaemia using an adaptation of flow cytometry based on the refractive properties of haemozoin, a pigment produced by malaria parasites after digestion of haemoglobin (Egan, 2008). These protocols represent versatile tools to explore the impact of host genetic variation on *Plasmodium* parasites. Understanding the mechanisms of host-parasite interactions at a molecular level may identify new targets for therapeutic intervention.

## Methods

### Ethics statement

The use of primary erythrocytes for the culture of Plasmodium falciparum was approved by the NHS Cambridgeshire Research Ethics Committee REC ref. 15/EE/0253 and the Wellcome Sanger Institute Human Materials and Data Management Committee HMDMC 15/076.

The use of human embryonic stem cell lines was approved by the Steering Committee for the UK Stem Cell Bank and for the use of Stem Cell Lines (ref. SCSC11-23) and the Wellcome Sanger Institute Human Materials and Data Management Committee. The Human Embryonic Stem cell lines (Shef 3 and Shef 6) were obtained from the Centre for Stem Cell Biology, University of Sheffield, Sheffield, UK.

The wild-type iPS cell lines were derived from fibroblasts and blood at the Wellcome Sanger Institute and obtained from the Wellcome Sanger HipSci collection.

The haemoglobinopathy iPS cell lines were derived from fibroblast samples obtained from the NIGMS human genetic cell repository of the Coriell Institute for Medical Research, USA: GM10796; GM03433.

### Reprogramming of induced pluripotent stem cell lines

Human IPS lines from haemoglobinopathy fibroblast samples acquired from the Coriell repository and from blood samples were derived and verified at the Wellcome Sanger Institute as described (Rouhani et al., 2014; Agu et al., 2015; Soares et al., 2016; Kilpinen et al., 2017). Briefly, 5x10^5^ cells were transduced with Sendai virus carriers of the Yamanaka factors: hOCT4, hSOX2, hKLF4 and hc-MYC overnight at 37^0^C in 5% CO_2_. After a medium change the next day, the cells were cultured for 4 days and from then on maintained in Stem Cell medium: advanced DMEM/F-12 (Gibco, UK) supplemented with 2 mM Glutamax (Gibco), 0.01% β mercapto ethanol (sigma), 4 nM human FGF-basic-147 (Cambridge Bioscience, UK) and 20% KnockOut serum replacement (Gibco, UK), changing medium daily. Ten to 21 days post-transduction, formation of pluripotent colonies was evident, the visible colonies were handpicked and transferred to 12 well plates with MEF feeders. Colonies were expanded into 6 well feeder plates and passaged every 5 to 7 days depending on confluence.

### Cell culture

All stem cell lines used in this study were cultured on feeder cells (irradiated mouse embryonic fibroblasts MEFS (Global Stem) in the Stem Cell medium described above. The cultures were kept at 37°C, 5% CO_2_ and medium was changed regularly. Pluripotent cells were passaged using 0.5 mM EDTA (Gibco, UK) and 10µM Rock inhibitor.

### Erythropoietic differentiation

Stem cells were taken off feeder cells with 0.5 mM EDTA (GIBCO) and seeded on gelatine-coated 10 cm plates pre-conditioned with MEF medium over-night and cultured in CDM-PVA supplemented with 12 nM hbFGF (Cell guidance systems, UK) and 10 nM hActivin-A (Source Bioscience, UK). CDM-PVA: 50% IMDM (Invitrogen), 50% advanced DMD-F12 (GIBCO) with 1g/l Poly(vinyl alcohol) PVA (SIGMA), Penicillin/Streptomycin 1x (GIBCO), 1-thioglycerol MTG (SIGMA), Insulin-Transferrin-Selenium 1x (ITS, Life Technologies), Cholesterol 1x (SyntheChol, SIGMA).

As a first step of differentiation, the cells were taken towards the mesoderm germline:

#### Mesoderm (2 days)

CDM-PVA medium supplemented with 5 nM hActivin-A and 2μM SU5402 (SIGMA)

#### Meso/Ery transition (8-12 days)

CDM-PVA medium supplemented with 20ng/ml bFGF, 10nM IL-3 (SIGMA), 10nM BMP4 (R&D Systems), 5μM SB431542 (SIGMA), 5μM CHIR99021 (Axon, The Netherlands), 5μM LY294002 (SIGMA).

During this stage, the detached cells are recovered, washed with PBS and transferred to the erythrocytic differentiation stage, performed in a basic erythrocytic medium (BEM): CellGRO SCGM (CellGenix, Germany) supplemented with ITS, cholesterol, 40ng/ml IGF-1 (Abcam), Penicillin/Streptomycin, 1µM 4-hydroxy 5-methytetrahydrofolate (SIGMA).

#### Ery I (4-5 days)

BEM supplemented with 10ng/ml IL-3, 50ng/ml SCF (Life Technologies), 1µM dexamethasone (SIGMA), 2U/ml EPO (SIGMA), 10ng/ml FLT3 (R&D Systems).

#### Ery II (4-5 Days)

BEM supplemented with 50ng/ml SCF, 1µM dexamethasone, 2U/ml EPO.

#### Diff Ery (minimum 4 Days)

BEM supplemented with 2U/ml EPO, 1µM Triiodo-L-Thyronine (T3, SIGMA)

### RNA extraction, qRT-PCR and microarrays

RNA was extracted using the Isolate II RNA Mini kit (Bioline, UK). 1-3 µg were reverse transcribed with a MuLV reverse transcriptase (Applied Biosystems, UK) using random primers (Bioline, UK). One µl of cDNA was specifically and quantitatively amplified using Biotool 2x SybrGreen qPCR master mix (Stratech, UK) following the cycling parameters established by the manufacturer on a light cycler 480 II (Roche) and using GAPDH as a control for normalisation. The primers used (IDT, Belgium) were:

**Table:** 

gene	forward primer	reverse primer	length (bp)
HbB	5’-gtctgccgttactgccctgtgg	5’-agcatcaggagtggacagatcc	136
HbA	5’-ggtgctgtctcctgccgac	5’-cctgggcagagccgtggctc	164
HbG	5’-cctgtcctctgcctctgcc	5’-cacagtgcagttcactcagc	140
HbE	5’-gctgccgtcactagcctgtg	5’-gcccaggatggcagagg	144
TAL1	5’-atgccttccctatgttcaccacca	5’-tgaagatacgccgcacaactttgg	108
Brach	5’-acaaagagatgatggaggaacccg	5’-aggatgaggatttgcaggtggaca	110
GATA1	5’-cctctcccaagcttcgtggaac	5’-caggcgttgcataggtagtggc	127
KLF1	5’-ccggacacacaggatgacttcc	5’-ctggtcctcagacttcacgtggag	114
GAPDH	5’-gcctcctgcaccaccaactgc	5’-ggcagtgatggcatggactg	102
OCT4	5’-ctgccgctttgaggctctgcagc	5’-cctgcacgagggtttctgc	134
NANOG	5’-ccagctgtgtgtactcaatgatag	5’-ctctggttctggaaccaggtcttc	123
GYPA	5’-ccactgaggtggcaatgcac	5’-cttcatgagctctaggagtggctgc	120
GYPC	5’-ggacattgtcgtcattgcaggtg	5’-gcctcattggtgtggtacgtgc	117
BSG	5’-ccatgctggtctgcaagtcagag	5’-cacgaagaacctgctctcggag	116
TfR	5’-gggctggcagaaaccttg	5’-cagttggagtgctggagact	145

For microarray analyses, RNA was extracted as above, the Illumina TotalPrep RNA amplification kit (Ambion Life technologies) was used to process the samples, and gene expression analysis was assessed on Illumina HumanHT-12v4 chips following the instructions of the manufacturer.

### Parasite culture

Fluorescent *P. falciparum* parasites were cultured in complete RPMI medium (GIBCO) at 2.5% haematocrit with O- RBCs sourced from NHSBT, Cambridge. Cultures were maintained at 37°C in malaria gas (1% O_2_, 3% CO_2_ and 96% N_2_).

### Fluorescent parasites

Parasites were engineered to express a variety of fluorochromes for detection at different wavelengths (Carrasquilla et al., 2020). The chosen fluorochromes: Midori-ishi cyan (excitation/mission: 472/495 nm, gift from Michael Davidson (Addgene plasmid # 54752; http://n2t.net/addgene:54752; RRID : Addgene_54752)), Kusabira Orange (excitation/emission: 548/559 nm, gift from Michael Davidson & Atsushi Miyawaki (Addgene plasmid # 54834; http://n2t.net/addgene:54834; RRID : Addgene_54834) (Karasawa et al., 2004)), tagBFP (Blue Fluorescent Protein excitation/emission 399/456 nm) and mCherry (excitation/emission 587/610 nm) were individually inserted into the XhoI/AvrII site of an *attP*-containing vector under regulation by the calmodulin promoter and bearing blasticidin resistance as a selection marker. The NF54 *attB* strain of *Plasmodium falciparum* was transfected with each fluorochrome vector together with an expression vector for Bxb1 integrase by pre-loading erythrocytes by electroporation with a BioRad gene pulser and incubating with percoll-purified late-stage parasites. The transfected cultures were maintained with daily medium change and transfectants were selected with blasticidin (2 µgr/ml) (Adjalley et al., 2010) after 2 days.

### Staining procedures

#### Cell labelling

*In-vitro*-differentiated erythroid cells were centrifuged (1100xg for 4’) and resuspended in 1 ml of Diff Ery medium containing 1 µM of a cell trace dye (ThermoFisher Scientific), either carboxy-DFFDA-SE ((Carboxylic Acid Diacetate) excitation/emission: 498/526 nm) or DDAO-SE ((9*H*-(1,3-Dichloro-9,9-Dimethylacridin-2-One-7-yl) β-D-Galactopyranoside) excitation/emission: 645/660 nm) dye for 1 h at 37^0^C. Cells were spun again and resuspended in 1ml dye-free medium and incubated for 30 minutes at 37^0^C. After a final spin, cells were resuspended in parasite culture medium at a concentration of 10^6^ cells per 75 µl.

#### Giemsa staining of slides

Cells, parasite cultures and invasion assays were stained with Giemsa for microscopic examination. Five µl of culture were dropped on a glass slide and spread with a pipette tip or smeared with a glass slide and dried. The slides were fixed with methanol for a few seconds, dried and incubated with 1x Giemsa stain solution for 5-10 minutes. The stain solution as drained away, the slides were washed with tap water and dried before microscopic examination.

#### Sybr Green staining of parasite DNA

Fixed culture samples were incubated in 100 µl of a 1X solution (1/10000 dilution) of Sybr Green (excitation/emission: 484/515 nm) nucleic acid gel stain 10000X (Invitrogen) for 30 minutes at 37^0^C. 200 µl of PBS were added to each sample before flow cytometry analysis using a 488 nm laser with a 530/40 nm bandpass filter.

### Invasion assays

*In-vitro*-differentiated labelled erythroid cells were counted and 75 μl containing 1 million cells were dispensed into a 96 well plate, including a well of cells alone for flow cytometry controls. Primary erythrocytes obtained from the blood bank were labelled in the same way, adding 75 µl at 5% haematocrit per assay.

Cultures of fluorescent parasites at a parasitaemia of 1.5 – 2% mature parasites were used to purify schizonts: 10 ml of culture were centrifuged (1100xg for 5’ brake 3), resuspended in 1 ml of medium and loaded onto a 63% Percoll cushion. Centrifugation at 1300xg for 11’ no brake separated the mature parasites at the Percoll interface. These were recovered, washed with parasite medium (as described in the parasite culture section) and resuspended in 3 ml of parasite medium. The parasite suspension, containing late-stage parasites was added to the cells in the 96 well plate at 75 μl/well.

One plate per time point was prepared and plates were placed in a gas chamber filled with malaria gas and left in a 37°C incubator for the appropriate length of time.

After the incubation time, the plate was removed, adding 200 μl of PBS/well and spinning at 1100xg 1’. Three μl were taken from the bottom of the wells and smeared on slides to be stained with Giemsa and the supernatant was removed. The pellets were fixed with 100 μl 4% paraformaldehyde for 20’, washed and resuspended in PBS to be analysed by flow cytometry.

### Flow cytometry

Expression of proteins on the membrane of stem cell-derived erythrocytes was measured using specific fluorochrome-tagged antibodies and quantitation by flow cytometry on a LSFORTESSA BD analyser using Flowjo V10.3 (Bcton Dickinson & Co, NJ) and FCSexpress 7. Antibodies:

CD71-APC (Allophycocyanin excitation/emission 594/660nm)Biolegend #334108

GYPA-PE (Phycoerythrin excitation/emission 565/574nm)Southern Biotech #9861-09

BSG-FITC (Fluorescein Isothiocyanate excitation/emission 495/525)MACS Miltenyi #130-104-489

ATP2B4-FITCLSBio #LS-C446496

A MoFlo flow cytometer (Beckman Coulter, USA) was adapted for detection of laser light depolarisation produced by parasite hemozoin (Frita et al., 2011). The Mo-Flo flow cytometer has a Z-configuration optics platform and is equipped with four solid state lasers (488nm, 561nm, 405nm, 640nm) spatially separated at the stream-in-air flow chamber with 488nm primarily assigned as the first laser. The laser power for 561nm, 405nm, 640nm were all set at 100mW and 488nm was set at 50mW. The laser 488nm, 561nm, 405nm, 640nm was used to excite the Cyan and PE, mCherry, BFP and DDAO respectively. Fluorescence emitted from Cyan, PE, mcherry, BFP and DDAO was collected using a 520/36nm, 580/30nm, 615/20nm, 447/60nm and 671/28nm band pass filter respectively. An optical modification was made on the primary laser detection pod so that the scattered light from 488nm laser light was split into two using a 50/50 beam splitter to measure the normal SSC (vertical) and depolarised SSC (Horizontal) by placing a polarizer (Chroma Technology Corp) with its polarisation axis horizontal to the polarisation plane of the laser light. Both SSC detectors have a 488/10nm band pass filter (**Supplementary Figure S2**). A total of 50000 events was acquired and analysed using Flojo V10.3 and FCSexpress 7.

Invitrogen Bigfoot cell sorter (Thermo Fisher Scientific, Inc.) was also used for the detection of laser light depolarisation produced by parasite hemozoin. The cell sorter is equipped with six spatially separated solid state lasers but only two of the lasers would be ON and used in the assay. The laser power for 488 nm was set at 125 mW and 640 nm was set at 100 mW. The laser 488 nm, 640 nm was used to excite Sybr Green and DDAO respectively. Fluorescence emitted from SYBR Green and DDAO was collected using a 507/19 nm and 670/30 nm band pass filter respectively. The instrument is also equipped with default polarisers at the 488nm laser light path which can be switched ‘ON’ during the analysis. This optic set up allows the measurement of normal SSC (488 SSC, Area Linear) and depolarised SSC (488 SSC Polar, Area Linear) (See figure below). A total of 20,000 events was acquired and analysed using FCSExpressv7 (*De Novo* Software, Inc.).

All samples are analysed on side and forward scatter to eliminate debris, then on pulse width to eliminate doublets and select the single cell population. Single cells are then analysed for fluorescence using the cell label and parasite fluorescence. The labelled non-infected cells are used to establish the depolarisation gate. The invasion assays are analysed in the same way, selecting the labelled population of cells and assessing depolarisation with the established gate. Quantification of the number of labelled erythroid cells containing metabolically active parasites in the total population of labelled cells reveals the % parasitaemia.

### Statistical analysis

Results are presented as means and standard deviation. The reported significance was calculated using a two-tailed unpaired Student’s T test analysis.

### Genome editing

Genomic modification to ablate the genes chosen for this study was performed in the RH1 cell line, as described previously (Yeung et al., 2017) and shown schematically in supplementary **Supplementary Figure S6**. Briefly, a CRISPR/Cas9 strategy was used targeting a critical exon in each gene (exon 5 in BSG and exon 11 in ATP2B4) for substitution with a selection cassette as depicted in **Supplementary Figure S6**. Pluripotent cells were nucleofected by electroporation using Amaxa4 following the manufacturer’s instructions. After two days of recovery, the puromycin resistance in the selection cassette was used to isolate correctly targeted clones which were examined for damage to the second allele by targeted sequencing as shown in **Supplementary Figure S7**.

### Microarray data analysis

All microarray datasets obtained as described in the RNA extraction, qRT-PCR and microarrays section were put through “neqc” background correction followed by quantile normalization using the limma R package (Ritchie et al., 2015). Inter-plate variation (batch effects) were adjusted using combat algorithm https://pubmed.ncbi.nlm.nih.gov/16632515/ [pubmed.ncbi.nlm.nih.gov]. Differential expression analysis was performed to obtain a subset of significant probes (those that change between two or more conditions), FDR adjusted P value of 0.05 was chosen as the cut-off using limma R package. Heatmaps were plotted using Complexheatmap R package https://pubmed.ncbi.nlm.nih.gov/27207943/[pubmed.ncbi.nlm.nih.gov]. The GSE63703 gene expression matrix from GREIN (https://shiny.ilincs.org/grein) was used to identify erythrocyte and erythroid progenitor specific genes.

## Results

### The differentiation protocol allows the *in-vitro* generation of erythroid cells from a wide variety of human stem cells

**Figure 1:**
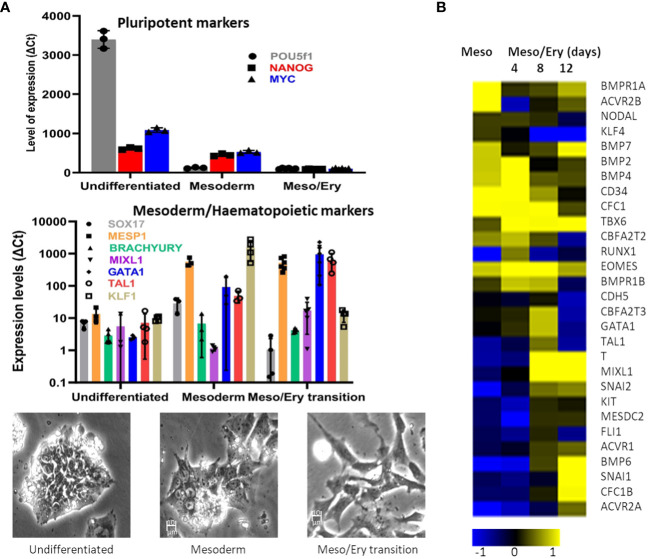
Differentiation of stem cells towards mesoderm. The RH1 cell line was differentiated and **(A)** Expression of pluripotency (POU5f2 (Oct 4), Nanog and c-myc), mesoderm (Sox17, MESP1 and Brach) and haematopoiesis (Mixl1, GATA1, Tal1, KLF1) markers was measured by qRT-PCR (at last 3 biological replicates are presented as a mean and standard deviation). Morphological changes of the differentiating cells are documented by light microscopy (100x) during regular culture of pluripotent cells (undifferentiated), after 2 days of mesoderm differentiation (mesoderm) and after 8 days of meso/ery differentiation (meso/ery transition). Cell differentiation is routinely followed by microscopic observation, of which the pictures presented are representative examples. **(B)** Transcriptome analysis of Mesoderm and haemato/erythropoiesis driver and signalling genes was performed by microarrays through the differentiation process. Meso, 2 days mesoderm differentiation; Meso/Ery, mesoderm/erythropoiesis transition after 4, 8 and 12 days of differentiation.

The main objective was to establish a stem cell differentiation protocol to generate erythroid cells able to support parasite invasion and growth, in sufficient numbers to allow parasite invasion assays to be performed (**Supplementary Figure S1**). With this in mind, we minimised cell manipulation and loss by avoiding the widely used embryoid body stage or co-cultures with other cell types and directing the cells towards the mesoderm path instead. This is achieved by two steps of specific cytokine combinations, based on the approach described by Vallier et al. (2009). First the cells are exposed to low levels of Activin A while inhibiting Fibroblast Growth Factor 2 (FGF2) to suppress neuroectoderm and then to a combination of Bone Morphogenetic Protein 4 (BMP4), FGF2 and inhibition of Activin A signalling with SB431542 to favour mesoderm differentiation over endoderm fate. In our protocol, interleukin 3 (IL-3) is added at this stage in order to direct the forming mesoderm towards haematopoiesis. As the cells start differentiating, qRT-PCR analysis shows decline of pluripotency gene expression while mesoderm markers increase (**Figure 1A**), before induction of the crucial transcription factors involved in driving the myeloid line of haematopoiesis towards megakaryocyte-erythroid progenitors (MEP). The modulation of gene expression is concomitant with a visible morphological change of the cells.

Microarray analysis confirmed the transition of the transcriptome from early mesoderm towards haematopoiesis through this stage of the differentiation process (**Figure 1B**). Expression of mesoderm specification genes (*eomes, BMP2/4, BMP receptor 1B (BMPR1B), Cripto (CFC1)*) became evident early during the mesoderm phase, peaking through the Meso-Ery transition, while the major drivers of haematopoiesis (*Brachyury (T), Tal1, GATA1and MIXL1*), peaked later. Based on the increased expression of haematopoietic genes towards the end of this stage of the protocol, the length of this step was set at 8 – 12 days. During this stage the cells spontaneously detach from the plates and can be harvested from the supernatant avoiding potentially damaging trypsinisation.

**Figure 2:**
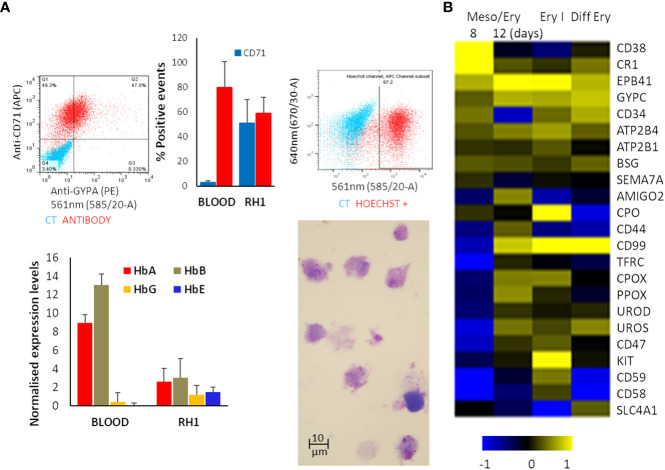
Erythropoietic stage of differentiation. The differentiated RH1 cell line was examined for **(A)** Expression of the main erythrocyte markers at the end of the differentiation process: Glicophorin A (GYPA) and transferrin receptor (CD71) as well as a comparison with primary erythroctyes (Blood), and DNA labelling with Hoechst33342 were assessed by flow cytometry (top plots); expression of the haemoglobins HbA, HbB, HbG and HbE was quantified by microarray presented as quartile normalised expression levels in both iPSC-derived erythroid cells (RH1) and primary erythrocytes (Blood) (bottom plot). Morphology of the cells is shown by Giemsa staining and light microscopy (1000x). **(B)** Microarray analysis of genes characteristic of haemato/erythropoiesis over time through the mesoderm/erythropoiesis transition (8 and 12 days of differentiation) step, the onset of erythropoiesis (Ery I: 4 days of the first step of erythropoiesis) and final erythrocytic differentiation (Diff Ery: 5 days of final erythropoietic differentiation) steps of the protocol.

The suspension of detached cells is then guided towards erythropoiesis by exposure to Erythropoietin (EPO) and IL-3 to drive differentiation into erythroblasts. Dexamethasone is added to halt the process at the erythroblast stage and improve the homogeneity of the culture. Cell numbers are increased by expanding the erythroblastic cells with Stem Cell Factor (SCF) and differentiation is completed by removing dexamethasone (**Supplementary Figure S1**). At the end of the differentiation process, analysis of the cells shows expression of the major erythrocytic marker Glycophorin A (GYPA) as well as adult haemoglobins A and B (**Figure 2A**). Most cells also express the transferrin receptor (CD71), and approximately 70% stain positively for nucleic acids (Hoechst33342), indicating that the cells still have some form of nucleus or nucleic acid content (nucleus or fragments thereof) (**Figure 2A**). Comparison of globin expression with primary erythrocytes shows that levels of the adult HbA and HbB are much lower and more variable and the iPSC-derived erythroid cells express high levels of foetal HbE and HbG (**Figure 2A**). These results indicate that the *in-vitro*-generated cells correspond to immature erythroid cells earlier in the erythropoietic differentiation pathway.

Microarray analysis of the transcriptome changes shows induction of major erythrocytic genes including structural proteins (EPB41, SCL4A1), functional proteins such as components of the haeme cycle (PO, CPOS, PPOX, UROS, UROD) and membrane transporters (ATP2B4, ATP2B1) as well as surface markers (GYPC, CD34, CD44, CD99, CD47) (**Figure 2B**). It is worth noting that induction of many erythrocytic genes occurs in the last stages of the meso/ery transition and the very early erythroid differentiation (Ery I). As erythropoiesis proceeds, the cells become less metabolically active, start extruding organelles and their RNA starts degrading. As a consequence, transcripts for some proteins, such as GYPA and TRFC (CD71) that we identify on the cell surface by flow cytometry (**Figure 2A**) are no longer detectable as we see in the microarrays. The full transcriptome changes through the differentiation process are shown in **Supplementary Figure S2**.

**Figure 3:**
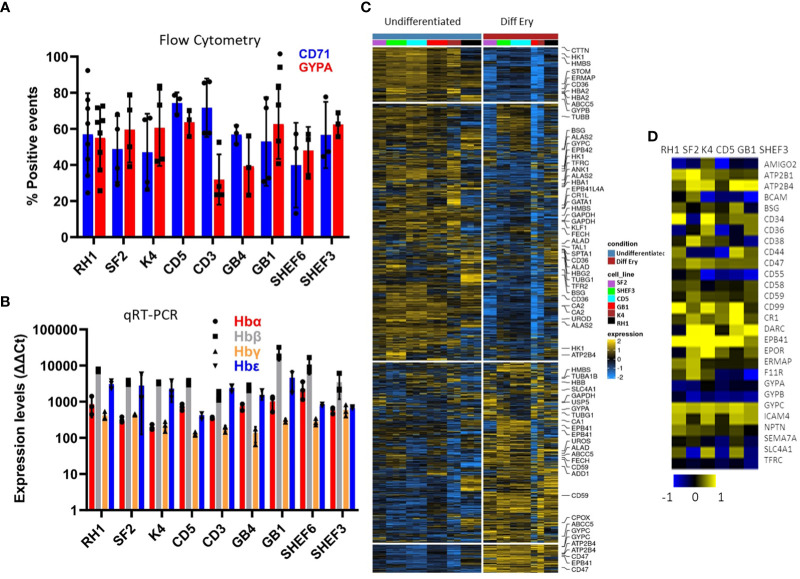
Characterisation of a variety of stem cell lines differentiated to erythrocytes *in-vitro* at the end of the differentiation protocol. **(A)** erythrocytic surface markers Glycophorin A (GYPA) and the transferrin receptor (CD71) by flow cytometry (average of at least 3 experiments, SD). **(B)** expression of the haemoglobins (adult A and B; foetal G and E) assessed by qRT-PCR (average of 3 experiments, SD). **(C)** Heatmap of the microarray analysis comparing the gene expression pattern in the iPS cell lines colour-coded on the right-hand side in Undifferentiated and differentiated (final erythropoietic phase) stages. The clusters group the subsets of genes from the highest levels of expression in pluripotency and lowest in erythroid differentiation on the top in descending and ascending order respectively towards the bottom **(D)** expression of the main erythrocyte surface receptors important for malaria parasites invasion in differentiated cells detected by microarray.

The versatility of the differentiation protocol was tested on a variety of cell lines of different origin, including human embryonic stem cell (hESC) lines (Shef3 and Shef6), as well as Induced Pluripotent Stem Cell (hIPSC) lines derived from both fibroblasts (RH1, SF2 and K4) and blood (CD3, CD5 and GB1, GB4). The efficacy of the process was assessed by expression of surface markers GYPA and CD71 by flow cytometry (**Figure 3A**) and the haemoglobin genes by qRT-PCR (**Figure 3B**). Though variations in the differentiation efficiency were observed particularly in the levels of GYPA, all the lines generated erythroid cells with distinct erythrocytic characteristics as revealed by the global comparison of gene expression using microarray analysis. As shown in **Figure 3C**, there is a dramatic change in gene expression between the pluripotent (undifferentiated) and erythroid (diff ery) states of all the iPS cell lines with a similar shift towards erythropoiesis.

Malaria parasites use a range of erythrocyte surface proteins as invasion receptors. *Plasmodium falciparum* in particular is well-known for its ability to use multiple invasion pathways and even switch between them to adapt to host polymorphisms or evade the immune response (Cowman et al., 2017). In this context, we assessed expression of genes reported to be important for invasion of erythrocytes (Bartholdson et al., 2013) using microarray analysis (**Figure 3D**). While some genes (e.g. *ATP2B4*, *CR1* and *GYPC*) were expressed in all the cell lines used, others (e.g. *CD55*) showed more variable expression at the transcript level. This highlights the need to assess the cell lines chosen for these types of studies in detail to ensure their suitability.

### Detection of haemozoin depolarisation accurately quantifies parasitaemia in invasion assays

**Figure 4:**
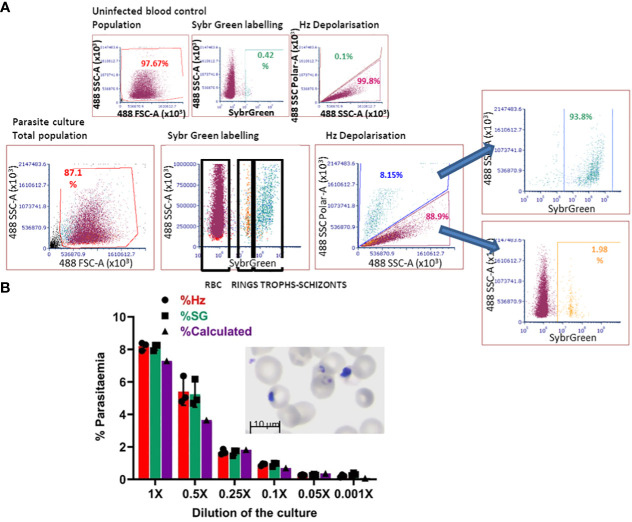
Haemozoin-based flow cytometry parasitaemia quantitation. **(A)** The population of uninfected erythrocytes (top left plot) is analysed for Sybr Green labelling (top middle) and Hz depolarisation (top right) to set the gates for defining positive and negative events. The parasite culture (Giemsa image) population (bottom left plot) is analysed with the set gates for Sybr Green labelling (middle plot) and Hz depolarisation (right plot). Sybr Green staining of the depolarising (far right-hand top plot) and non-depolarising events (far right-hand bottom plot). Plots are representative of 3 experiments. **(B)** The parasite culture (Giemsa image) was serially diluted with uninfected erythrocytes. Parasitaemia was quantified by Hz depolarisation, Sybr Green labelling and calculation from manual counting of the original slide.

The *in-vitro*-derived erythroid cells maintain some nucleic acid content, revealed by Hoechst33342 staining (**Figure 2A**), making the use of DNA labelling to quantify parasitaemia incompatible. To circumvent this problem, we adapted a flow cytometry approach to detect haemozoin (Hz) (**Supplementary Figure S3A**), a product of haemoglobin digestion by the parasite that has the property of depolarising light. We first assessed whether depolarisation caused by Hz reflects parasitaemia in our system, by labelling a culture of parasites (**Figure 4** Giemsa image, counted at 7.3% parasitaemia) with Sybr Green (SG, excitation/emission: 484/515 nm) and comparing direct parasite detection with Hz quantification (**Figure 4A**). The flow cytometry gates were set up with uninfected blood (**Figure 4A** top plots, no positive events in SG labelling nor Hz depolarisation). The parasite culture was analysed in the same way applying the established gates (**Figure 4A**), that revealed several SG-positive populations (**Figure 4** bottom middle plot: Sybr Green labelling) of increasing intensity, deemed to reflect rings and brighter mature stages. The depolarisation gate (**Figure 4A** Hz depolarisation plot) detected 8.15% positive events, which corresponded well with the 8.14% of SG-positive cells and the 7.3% manual count of the corresponding Giemsa-stained slide (**Figure 4A** image). Examining the depolarising population for SG staining (**Figure 4A** far right top plot) confirmed that 93.8% of this population corresponds to SG-labelled parasites. The non-depolarising population stained with SG (**Figure 4A** far right bottom plot) contains mostly uninfected erythrocytes and 1.98% of low intensity SG-positive cells, corresponding to small rings that have not yet accumulated enough Hz to be identified by depolarisation.

With the aim of assessing the detection capacity and limits of Hz depolarisation, the same parasite culture from **Figure 4A** was diluted by repeatedly adding an equal volume of a preparation of uninfected erythrocytes at the same haematocrit (2.5%) to create a serial dilution of the parasite culture (**Figure 4B**). Parasitaemia was quantified by SG staining and Hz depolarisation (**Figure 4B**) and compared with the calculation from the manual counting of the original Giemsa slide. A good correspondence between the three methods was observed at the decreasing levels of parasitaemia. The detection limit was found at a dilution of 0.05x that corresponded to a calculated 0.365% parasitaemia, beyond which the quantification becomes unreliable for both, Hz depolarisation and SG staining, remaining at a level between 0.2 and 0.3%.

**Figure 5:**
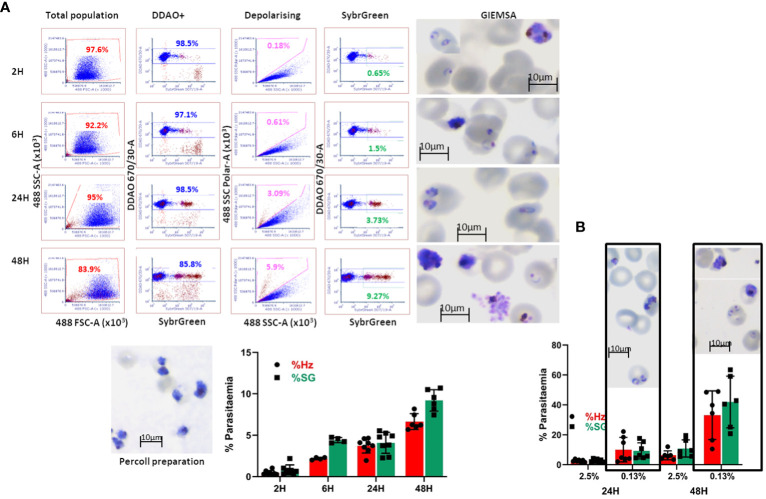
Invasion assay time course of parasitaemia. **(A)** The purified parasites (Percoll preparation) are cultured with DDAO-stained erythrocytes and incubated for the reported times. The culture populations (left plots) are analysed for DDAO to identify the target cell population (DDAO+). Parasitaemia is quantified by Haemozoin depolarisation (Depolarising) as well as Sybr Green labelling (SybrGreen) using gates established with uninfected DDAO-labelled erythrocytes. Giemsa-stained slides are made at each time point before fixing the cultures. The overall results are shown as mean and SEM of at least 4 biological replicates. **(B)** Comparison of infection in routine (2.5%) and low (0.13%) haematocrit. Erythrocytes were labelled with DDAO and invasion assays set at the indicated haematocrit for 24 and 48 hours. Parasitaemia was assessed by Hz and SG labelling (average and SD of at least 5 experiments) and parasites were visualised by Giemsa staining.

In order to quantify *P. falciparum* parasitaemia, we designed invasion assays as shown in **Supplementary Figure S3B**, in which target cells are co-cultured with purified late-stage parasites and incubated in a 96 well plate for set times and analysed by flow cytometry. This approach was first evaluated using as target cells primary erythrocytes labelled with the membrane dye DDAO (excitation/emission: 645/660 nm) to specifically quantify invasion of the target cells. In the first instance, we followed the time course particularly of the early hours of invasion in these assays (**Figure 5A**). After eliminating debris and doublets the population of target cells is identified by the membrane staining (**Figure 5A** DDAO+) and this population is examined for depolarisation. To confirm that the depolarising events are indeed cells infected with the parasites, the assays were stained with Sybr Green (SG) and the DDAO+ population was also examined for SG signal. A sample of the invasion assay culture was smeared on a slide and stained with Giemsa to check the stage of the parasites. Parasitaemia can be detected as soon as 2 hours post-invasion, though the increase observed at 6 hours indicates that invasion still occurs beyond the 2-hour time point. The difference between Hz and SG quantification 6 hours post-infection shows that accumulation of Hz is still below detection in about half of the parasites. At 24 hours, the levels of parasitaemia are maintained, with detection by Hz reaching similar levels as SG, confirming parasite growth and metabolic activity. Parasitaemia increases at 48 hours and an underestimation of Hz quantification compared to SG indicates that reinvasion has occurred with small parasites appearing in the assays as shown in the Giemsa slides. On this basis the time point chosen for erythroid cell assays were 18 hours to assess invasion and 42 hours to evaluate development. These results show that Hz detection is an accurate and effective way to detect malaria parasites and quantify parasitaemia. It is particularly useful when parasites cannot be stained or labelled with routine methods or when staining is impractical, being also applicable to any parasite strain or species.

The differentiation protocol can generate substantial numbers of cells (10-20 million from a 10 cm plate of pluripotent stem cell cultures) however this not sufficient to emulate routine erythrocyte cultures. In order to assess feasibility of this approach for malaria studies, the haematocrit of regular cultures (2.5%) was reduced to as low as 1/20 (approximately 0.13%). Comparison of invasion at these levels (**Figure 5B**) showed that invasion at the lower haematocrit is higher as would be expected, and this is more noticeable at the second time point. The similar parasitaemia quantified by Hz and SG shows that the events detected are indeed infected erythrocytes and that the assays are effective at lower haematocrit. Furthermore, the higher levels of parasitaemia facilitate assessment of invasion differences between cells of different origin or characteristics.

### Stem cell-derived erythroid cells support invasion by *Plasmodium falciparum*

The accurate quantification of the capacity of stem cell-derived erythroid cells to support *P. falciparum* infection, depends on the effective exclusion of non-invading parasites. On one hand, it is crucial to label the target cells as we show in the previous section, to only quantify invasion in the *in-vitro*-derived cells. Additionally, in order to ensure accurate quantification of invading parasites we engineered fluorescence-expressing parasites to enable their localisation on the flow cytometry plot and clearly separating the non-invading parasite population from the population of labelled erythroid cells. For this, we used the NF54 *P. falciparum* strain to create parasite lines expressing fluorochromes of different wavelengths (Carrasquilla et al., 2020) to identify the free parasites and ensure Hz quantification only in the target cells to reflect the % parasitaemia.

Several fluorescent parasite lines and membrane dies to label *in vitro*-derived erythroid cells from the model cell line RH1, were tested to find the combinations leading to the clearest parasitaemia determination and show the versatility of this system. In the first instance, parasites expressing the Midori-ishi cyan fluorochrome (**Supplementary Figure S4A**, top left) which has a similar range of excitation/emission wavelengths (472/495 nm) as SG were used. Uninfected *in-vitro*-differentiated erythroid cells were labelled with DDAO (**Supplementary Figure S4A**, top middle) alone were examined for Hz depolarisation to establish the gates as in the previous section (**Supplementary Figure S4A**, top right). The invasion assays of Midori-ishi-parasites with DDAO-erythroid cells were assessed in the same way, applying the established gates to identify the target erythroid cell population (**Supplementary Figure S4A** bottom left plot) and quantify the proportion of Hz-positive events within the population of labelled cells (**Supplementary Figure S4A**, bottom right plot), which corresponds to infected erythroid cells. Overlay of the depolarising population from the invasion assays over non-infected cells and free parasites confirms that the Hz-containing events correspond to stem cell-derived erythroid cells infected with the parasite (**Supplementary Figure S4A**, far right panel).

An alternative combination was tested, using erythroid cells labelled with DFFDA (excitation/emission: 498/526 nm) and parasites expressing mCherry (excitation/emission: 587/610 nm) which also enabled separating the populations of labelled erythroid cells and purified parasites allowing accurate quantification of parasitaemia (**Supplementary Figure S4B**). The preferred combination was labelling the target cells with DDAO and culturing with parasites expressing tag-BPF (excitation/emission: 399/456 nm) because of the strong fluorescence and the wider separation of their spectra (**Supplementary Figure S4C**). This was the chosen combination for all following experiments.

Because the quantification of depolarising events is applied to the whole population of labelled cells, this proportion reflects the parasitaemia of the culture. Since haemozoin is the metabolic product of the parasite’s digestion of haemoglobin (Frita et al., 2011), it only accumulates in the food vacuole as a result of parasite metabolism, and therefore its detection reflects live, active parasites in the differentiated erythroid cells. Some events can be missed if Hz accumulation has not reached detectable levels by the time of measurement.

**Figure 6:**
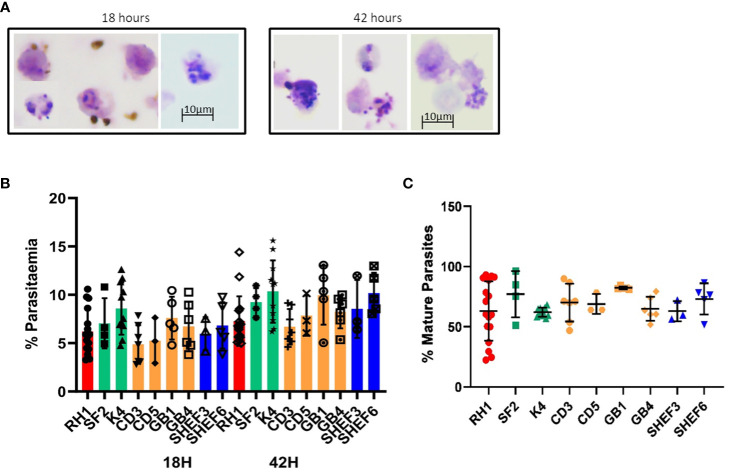
Invasion of in-vitro-generated erythrocytes with *P. falciparum*. **(A)** Giemsa-stained slides of invasion assays at 18h and 42h of culture (representative examples of routine imaging). **(B)** Quantification of invasion by Hz flow cytometry of a variety of differentiated cell lines: iPS cell lines derived from fibroblasts (red, green) and blood (yellow) and human ESC lines (blue) at two time points of culture, 18h (invasion) and 42 hours (development) (results are shown as mean of at least 3 experiments and SD). **(C)** quantification of mature parasites percentage at 42 hours (mean of at least 3 experiments, SD).

Invasion assays with the *in-vitro*-generated erythroid cells showed successful invasion upon examination of Giemsa-stained slides (**Figure 6A**). Analysis of 50.000 events by flow cytometry showed erythroid cells differentiated from all nine stem cells lines tested were effectively invaded by *P. falciparum* (**Figure 6B**). At 18 hours post-invasion, the parasitaemia ranged between 5 and 8% and at 42 hours parasitaemia rose in all cell lines to 7–10%, likely reflecting the growth of small parasites that went undetected at the 18-hour timepoint (**Figure 4B**). The level of infection detected in erythroid cells is lower than the equivalent primary erythrocytes shown in **Figure 5**, particularly at the second time point, which might reflect the difference between native and model cells, particularly in terms of globin expression.

The concomitant increase in Hz intensity with life cycle progression is a useful tool to measure parasite growth (Frita et al., 2011; Rebelo et al., 2013). Indeed, synchronised parasite cultures show a clear shift in Hz signal intensity as they progress from rings to schizonts (**Supplementary Figure S5A**). The difference in Hz intensity between the schizont and ring stages (**Supplementary Figure S5A**) was used to establish a gate (M1) to quantify the proportion of mature parasites in the invasion assays in both primary erythrocytes and *in vitro*-derived erythroid cells (**Supplementary Figure S5B**). Applying the same gate to the assays with the *in-vitro*-generated erythroid cells, detected levels of mature parasites between 60 and 80% at the 42-hour time point (**Figure 6C**), indicating parasite development.

### Genome editing of human stem cells reveals the role of specific genes in malaria invasion

**Figure 7:**
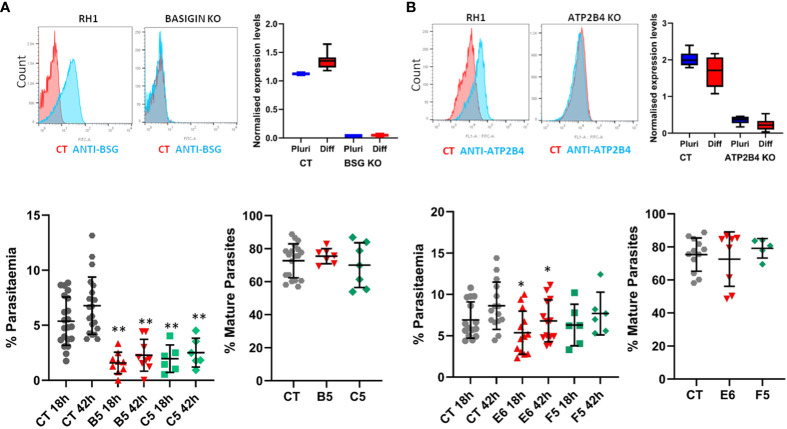
Gene editing in stem cells to study the impact of specific genes on malaria infection. Two genes of interest were deleted in the RH1 cell line using CRISPR/Cas9 and clones were isolated: **(A)** Basigin and **(B)** ATP2B4. Top panels: Expression of the protein corresponding to the deleted gene was measured on the cell surface with specific antibodies by flow cytometry (representative example of 3 experiments) and confirmed by microarray analysis of the cell lines transcriptomes in undifferentiated and differentiated states, presented as quantile normalised log2 expression values (average and SD of at least 3 data sets). Bottom panels: invasion of the parental line RH1 (CT) compared to the Basigin KO clones B5 and C5 **P<.001 and ATP2B4 KO clones E6 and F5 *P<.05, as well as percentage of mature parasites at 42 hours. (average and SD from at least 5 experiments).

The potential to derive edited erythrocytes by introducing targeted modifications in the stem cell lines was tested using CRISPR/Cas9 technology in the control line RH1 (**Supplementary Figure S6**). Two target genes were chosen, Basigin (*BSG*, CD147) which is known to be a universal receptor for *P. falciparum* invasion (Crosnier et al., 2011) as a proof of principle, and *ATP2B4* (*PMCA4*) since natural variation in this gene has been correlated to resistance to severe malaria (Timmann et al., 2012). The edited clones were genotyped (**Supplementary Figure S7A**) and verified by sequencing (**Supplementary Figure S7B**) confirming small deletions in the critical exon that generate a stop codon downstream. The lack of protein expression was confirmed with specific FITC-labelled antibodies by flow cytometry and microarray analysis of the pluripotent and differentiated cells (**Figure 7A**).

When challenged with the parasites, erythroid cells differentiated from two independent Basigin-null cell lines (B5 and C5) showed strongly decreased invasion compared to the parental non-edited RH1 cells (**Figure 7B**, left), consistent with the role of Basigin as an essential receptor for *P. falciparum* (Crosnier et al., 2011). The proportion of mature parasites after 42 hours of culture was similar to the control RH1 cells, indicating that the few parasites that invaded the modified cells could achieve some growth. Deletion of ATP2B4 (**Figure 7A**, right) showed a tendency to lower invasion levels compared to RH1 cells, but this decrease was significant for only one clone (**Figure 7B**, right). The proportion of mature parasites in the ATP2B4 KO cultures at the 42-hour time point was similar to that observed in the control RH1 line, indicating that disruption of this gene has a minor effect on the development of the parasite.

### Reprogrammed IPS lines from haemoglobinopathy patients show a deficiency in parasite invasion

In order to assess the potential of this technology to study the genomic context and particular genotypes from specific individuals, we chose α-thalassemia as a human trait strongly associated with resistance to malaria infection. We chose α-thalassemia major, in which all four α-globin genes are impaired in order to have a strong phenotype and also because this type is not viable, meaning that primary blood samples cannot be obtained.

**Figure 8:**
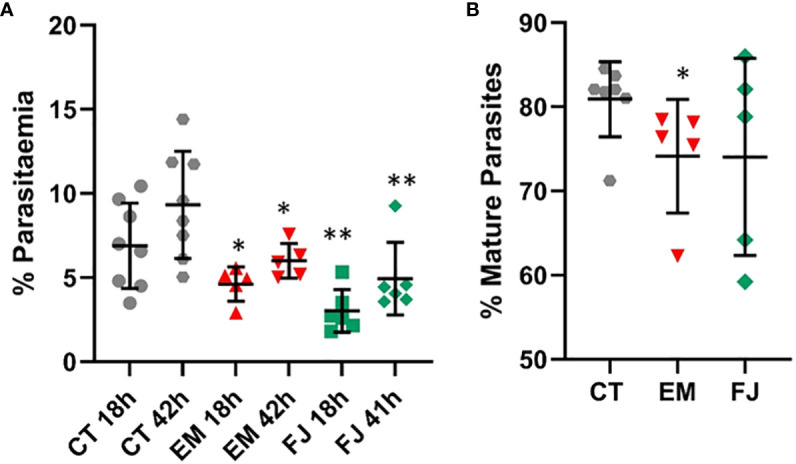
Invasion of reprogrammed IPS cells from patients with α-thalassemia haemoglobinopathy. Fibroblasts from α-thalassemia major samples were reprogrammed to IPS cell lines (EM and FJ) that were differentiated to erythroid cells and exposed to *P. falciparum* to assess **(A)** invasion in parallel to control cell lines (CT) and **(B)** percentage of mature parasites at 42 hours (results are presented as mean and SD of at least 3 independent experiments) *P<.05; **P<.005.

Induced Pluripotent Stem cell lines derived from fibroblast samples with α-thalassemia major (HbBart) haemoglobinopathies (EM andFJ) were differentiated in parallel with our reference iPSC lines and exposed to fluorescent parasites in our invasion assays. **Figure 8A** shows a significantly decreased invasion in both thalassaemic cell lines compared with the control lines. Though at the second time point 42 hours after invasion parasitaemia was significantly increased in the haemoglobinopathy cells, this remained significantly below the levels observed in the control wild type lines. Interestingly, while the parasitaemia at 42 hours in the haemoglobinopathy cells was significantly reduced compared to that observed in wild type cells (**Figure 8A**), the proportion of late stage parasites in the cultures of the haemoglobinopathy lines at the 42-hour time point was not significantly different from the wild type lines. Indeed only a marginally significant reduction was observed in one of the cell lines tested (**Figure 8B**). This suggests that the main impact of α-thalassemia major on malaria infection is at the invasion stage.

## Discussion

This work presents a protocol that effectively differentiates a variety of human stem cell lines towards erythropoiesis generating cells able to support *Plasmodium falciparum* infection. A total of 9 stem cell lines of diverse origin were studied, showing that while differentiation efficiency does vary between lines, they all generate erythroid cells as demonstrated by upregulation of erythrocytic genes and expression of erythrocytic proteins. Despite enucleation being notoriously difficult to achieve *in-vitro* (Lu et al., 2008; Hirose et al., 2013) we observed levels of 20-30%, bearing in mind that cells with positive nucleic acid staining include those with nuclear fractionation, incomplete nuclear extrusion (**Figure 1B**) and remnant nucleic acid content. Importantly, stem cells differentiated with this protocol are capable of supporting invasion by *P. falciparum* without the need to sort or purify the differentiated cells.

During the blood cycle, haemoglobin is the main source of amino acids for the parasite’s metabolic needs. Degradation of haemoglobin releases free haem, which represents a major toxic insult to the parasite. In a detoxification mechanism the oxidised iron group is compacted into an insoluble crystalline form: β-hematin or haemozoin (Hz) and stored in the food vacuole (Egan, 2008), becoming a distinct feature of intra-erythrocytic Plasmodium parasites (Rebelo et al., 2013). We show in this work that detecting Hz by flow cytometry is an effective and accurate method of quantifying parasitaemia. Comparing parasitaemia quantification by Hz with Sybr Green (SG) DNA staining of the parasites confirms the depolarising events as parasites and the accuracy of the method. Intracellular Hz is known to accumulate in the parasite, becoming detectable after 6 hours post-invasion to 70-80% by dark-field microscopy (Delahunt et al., 2014). The sensitivity of flow cytometry can detect Hz earlier as shown in **Figure 5**, and though beyond the scope of this work, this capability has the potential to decipher effects on early development with our approach. During progression of the blood cycle, both Hz crystal size and number increase in the parasites (Chen et al., 2019), which makes Hz intensity an effective measure of parasite development compared to DNA labelling intensity which only increases with parasite replication.

The use of schizonts for the assays ensures quick invasion after the co-culture is set up, but complete synchrony is difficult to achieve, resulting in some contribution of less mature parasites in the schizont preparations, which is reflected in the increase of Hz-positive events at 42 hours. It is also possible that parasite growth in the erythroid cells is slower due to the overall lower levels of globin expression, which could explain the lower parasitaemia observed in comparison to primary erythocytes in the same conditions. The use of fluorescent parasites constitutes an additional control for the accurate quantification of parasitaemia, however detection of Hz makes it possible to perform this type of studies with any parasite strain as demonstrated in **Figures 4**, **5**. It is useful to use fluorescent control parasites in parallel to non-fluorescent ones to ensure exclusion of the free parasite population from quantification.

The possibility to genetically manipulate genes implicated in malaria infection was demonstrated by deletion of *Basigin*, which resulted in a dramatic decrease of infection as expected given the known role of this protein in invasion (Crosnier et al., 2011). The low levels of invasion detected are likely the result of true invasion because the accumulation of Hz reflects live parasites and also because the very few invasion events show signs of development at the second time point. While it is widely accepted that Basigin is a crucial receptor for *P. falciparum* invasion, the high sensitivity and additional controls included in the strategy used here allowed high accuracy in the quantification of invasion. Our observations are consistent with other studies using blocking antibodies showing residual invasion in the laboratory (Crosnier et al., 2011) and variable levels of inhibition of field isolates *in ex-vivo* invasion assays (Moore et al., 2021). Natural variants in ATP2B4 have been associated with resistance to malaria in various studies (Ndila et al., 2018; Malaria Genomic Epidemiology N, 2019), but the mechanism of protection is not known. A number of variant SNPs have been identified in this gene, mostly in Linkage Disequilibrium (LD), and though it is not clear whether all these SNPs play a role in protection against severe malaria, one of them was shown to disrupt a GATA-1 site in the promoter of the gene (Lessard et al., 2017). As a consequence, expression levels of the protein are reduced giving rise to changes in erythrocyte parameters such as mean corpuscular haemoglobin concentration (MCHC) and size. ATP2B4 is the main membrane Calcium ATPase of erythrocytes that removes calcium from the cytosol to maintain the low levels necessary for calcium-dependent signalling to occur (Dalghi et al., 2013). A role of calcium in the invasion process of *P. falciparum* has been suggested (Gao et al., 2013; Volz et al., 2016) and it is also possible that impairment of calcium homeostasis affects survival and development of the parasite in the erythrocyte (Gazarini et al., 2003; Lessard et al., 2017; Zambo et al., 2017). However, a knock-out of *ATP2B4* in our system did not show a major effect on *P. falciparum* invasion or growth, though a tendency towards a reduction in both parameters was observed. A compensatory effect of ATP2B1 (*PMCA1*), which represents 20% of erythrocytic Calcium ATPases, could explain the minimal effect of deleting *ATP2B4*. The ubiquitous expression of *ATP2B4* throughout the body, could also imply other effects on the disease, such as the interaction of infected erythrocytes with endothelial cells or with the brain, as has been suggested (Timmann et al., 2012).

We further demonstrate the adaptability of this strategy by reprogramming iPS cells from haemoglobinopathy patients, a trait known to confer protection against malaria. Alpha-thalassemia results from a variety of large deletions affecting one or more of the duplicated alpha globin genes and the severity of the disease depends on how many of the four genes are affected. Loss of all 4 α-globin genes, known as α-thalassemia major, can occur in the common South East Asian deletion, leading to the lethal HbBarts hydrops foetalis. Alpha-thalassemia major was chosen for these studies because of the extreme phenotype and because primary erythrocytes with this genotype are unavailable to perform laboratory assays, thus highlighting the advantages of stem cell technology. Both reprogrammed cell lines are null for alpha globin, presenting –SEA/–SEA (GN03433) (Ho et al., 2007) and –SEA/–Fil (GM10796) (Hong et al., 2017) genotypes. When differentiated, both cell lines showed a significantly reduced ability to support *P. falciparum* infection, consistent with reported effects of haemoglobinopathies on malaria (Taylor et al., 2013; Pathak et al., 2018). Though several mechanisms have been proposed, it is still unclear how haemoglobin deficiencies impact the parasite and their study is complicated by the variety of genetic changes underlying these traits as well as the difficulty in obtaining samples of primary erythrocytes. It is known that the imbalance in the synthesis of globin chains in alpha and beta thalassemias result in impairment of the assembly of haemoglobin tetramers. This leads to the formation of haemoglobin precipitates (Heinz bodies), which together with the increased hydration occurring in α-thalassemias impair erythrocyte deformability (Huisjes et al., 2018). It was shown that erythrocyte deformability is lower in samples of α-thalassemia traits in which 2 alpha globin genes are inactivated and the decrease is much stronger in Haemoglobin H disease in which 3 alpha globin genes are missing. It is reasonable to predict an even greater deformability defect in the total absence of haemoglobin alpha of the cell lines used here. Furthermore, this decrease in deformability was directly corelated to decreasing *P. falciparum* invasion (Bunyaratvej et al., 1992) which is consistent with the observations using our haemoglobinopathy lines that it is mainly the invasion process that is impacted by these genotypes. Additionally, it was shown that *P. falciparum* parasites produce significantly lower numbers of merozoites in alpha and beta thalassemia trait cells, correlating with the MCHC and mean corpuscular volume (MCV) of these cell types (Glushakova et al., 2014).

This novel application of stem cell technology for the study of malaria presents a new and exciting avenue to understand the impact and mechanisms of complex genetic traits and human genomic variation in their full genomic context. As any model of study, there are aspects that need to be taken into account when taking advantage of this system, such as the immaturity of erythroid cells generated, the number of cells that can be obtained and their heterogeneity. However, it is the only option to genetically engineer erythrocytes without depending on particular cell lines as well as generate them from patients or samples with specific characteristics. Though in this work we aimed at developing a strategy with minimal handling that can be potentially scaled up for screening purposes, it can also be easily adapted to more detailed studies by the possibility of labelling receptors of interest or sorting the erythroid cells from the *in-vitro*-differentiated population as well as assessing any parasite strain and using tightly synchronised parasites. The potential to use existing resources of banked available cell lines as well as reprogramming iPS lines from easily obtainable blood samples from patients or individuals with specific genotypes offers access to the study and preservation of a wide range of genetic characteristics. This approach is a powerful tool for the understanding of this disease, offering a wide range of applications from functional and mechanistic studies to potential identification of therapeutic targets.

## Data availability statement

The microarray data presented in this study are deposited in the GEO repository, accession number GSE245735: https://www.ncbi.nlm.nih.gov/geo/query/acc.cgi?acc=GSE245735.

## Ethics statement

Ethical approval was not required for the studies on humans in accordance with the local legislation and institutional requirements because only commercially available established cell lines and samples from repositories were used.

## Author contributions

AP: Conceptualization, Data curation, Formal analysis, Investigation, Methodology, Supervision, Validation, Visualization, Writing – original draft, Writing – review & editing. BN: Conceptualization, Investigation, Methodology, Writing – original draft. KM: Data curation, Formal analysis, Investigation, Methodology, Visualization, Writing – original draft. MK: Investigation, Methodology, Visualization, Writing – original draft. CA: Investigation, Methodology, Writing – original draft. FR: Investigation, Methodology, Writing – review & editing. RM: Investigation, Methodology, Visualization, Writing – review & editing. FL: Investigation, Validation, Writing – review & editing. HP: Data curation, Investigation, Methodology, Writing – review & editing. JR: Conceptualization, Funding acquisition, Resources, Writing – review & editing.
