# Supplementary material for: Novel stem cell technologies are powerful tools to understand the impact of human factors on Plasmodium falciparum malaria
Source: Front Cell Infect Microbiol. 2023 Dec 19;13:1287355. doi: 10.3389/fcimb.2023.1287355 (PMC10762799; doi:10.3389/fcimb.2023.1287355)
Supplement: Supplementary file 1 [file Presentation_1.pptx]

## Slide 1
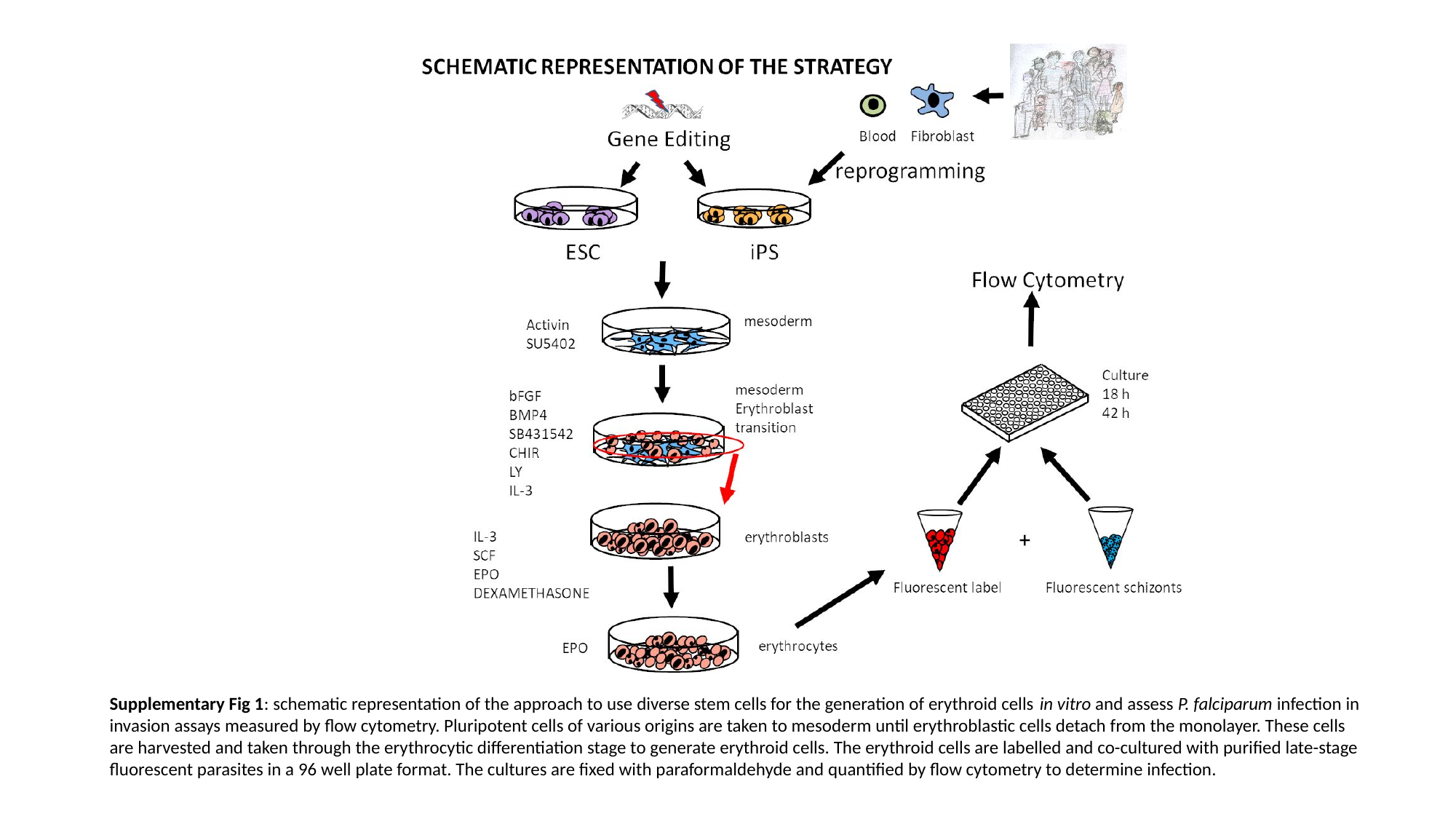

Supplementary Fig 1: schematic representation of the approach to use diverse stem cells for the generation of erythroid cells in vitro and assess P. falciparum infection in invasion assays measured by flow cytometry. Pluripotent cells of various origins are taken to mesoderm until erythroblastic cells detach from the monolayer. These cells are harvested and taken through the erythrocytic differentiation stage to generate erythroid cells. The erythroid cells are labelled and co-cultured with purified late-stage fluorescent parasites in a 96 well plate format. The cultures are fixed with paraformaldehyde and quantified by flow cytometry to determine infection.

## Slide 2
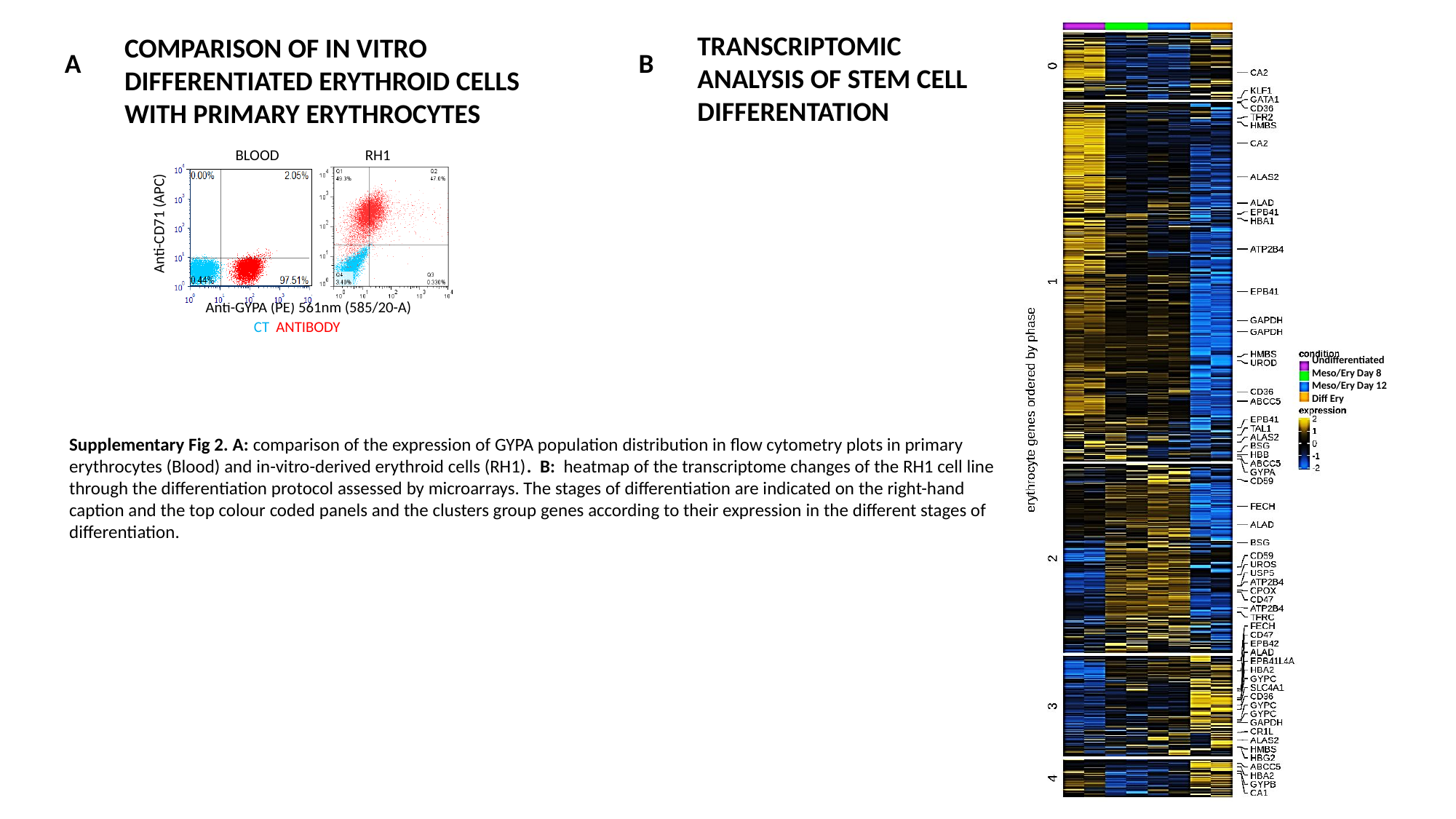

Undifferentiated
Meso/Ery Day 8
Meso/Ery Day 12
Diff Ery
TRANSCRIPTOMIC ANALYSIS OF STEM CELL DIFFERENTATION
COMPARISON OF IN VITRO DIFFERENTIATED ERYTHROID CELLS WITH PRIMARY ERYTHROCYTES
A
B
BLOOD RH1
Anti-CD71 (APC)
Anti-GYPA (PE) 561nm (585/20-A)
CT ANTIBODY
Supplementary Fig 2. A: comparison of the expression of GYPA population distribution in flow cytometry plots in primary erythrocytes (Blood) and in-vitro-derived erythroid cells (RH1). B: heatmap of the transcriptome changes of the RH1 cell line through the differentiation protocol assessed by microarrays. The stages of differentiation are indicated on the right-hand caption and the top colour coded panels and the clusters group genes according to their expression in the different stages of differentiation.

## Slide 3
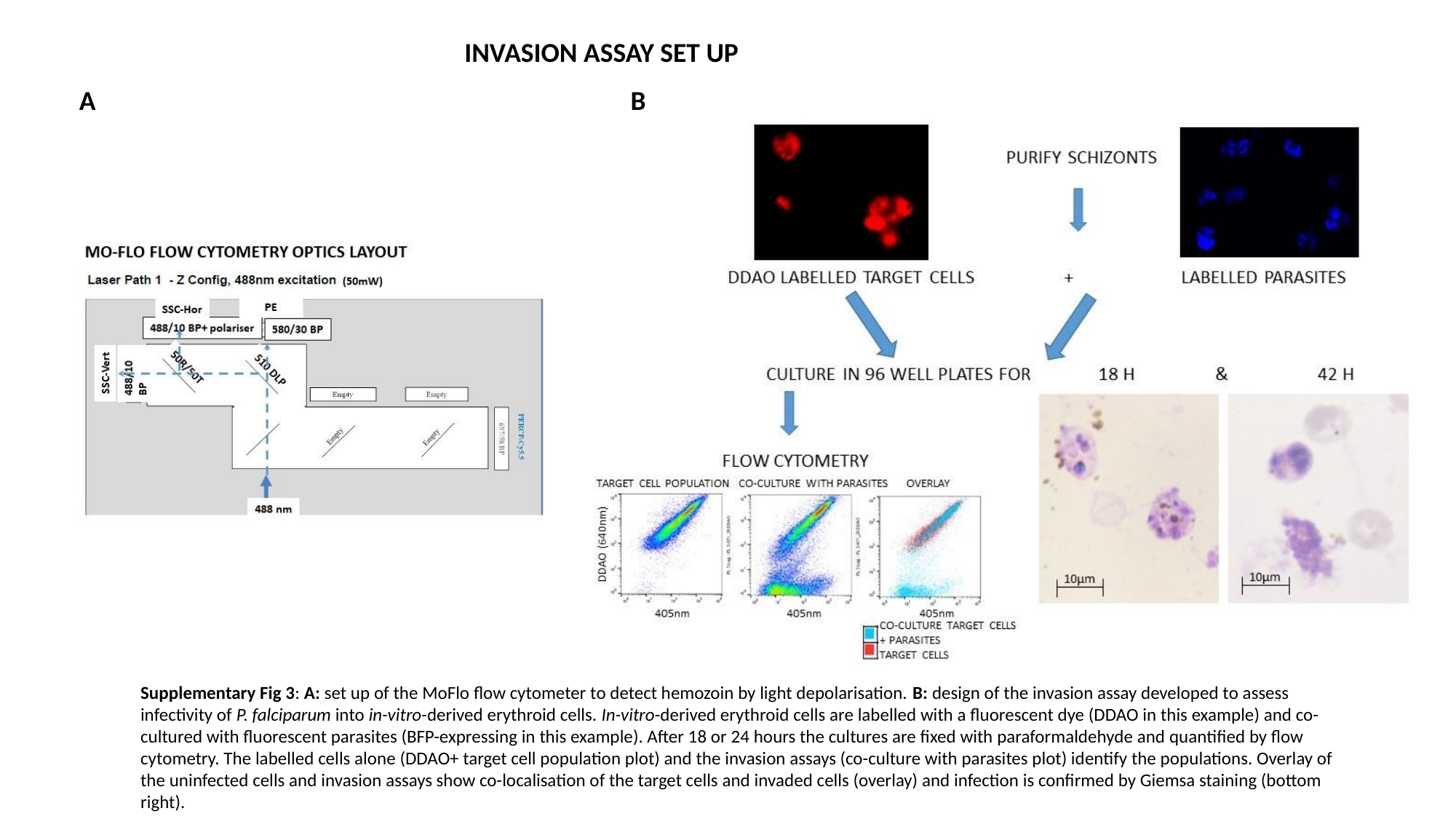

INVASION ASSAY SET UP
A
B
Supplementary Fig 3: A: set up of the MoFlo flow cytometer to detect hemozoin by light depolarisation. B: design of the invasion assay developed to assess infectivity of P. falciparum into in-vitro-derived erythroid cells. In-vitro-derived erythroid cells are labelled with a fluorescent dye (DDAO in this example) and co-cultured with fluorescent parasites (BFP-expressing in this example). After 18 or 24 hours the cultures are fixed with paraformaldehyde and quantified by flow cytometry. The labelled cells alone (DDAO+ target cell population plot) and the invasion assays (co-culture with parasites plot) identify the populations. Overlay of the uninfected cells and invasion assays show co-localisation of the target cells and invaded cells (overlay) and infection is confirmed by Giemsa staining (bottom right).

## Slide 4
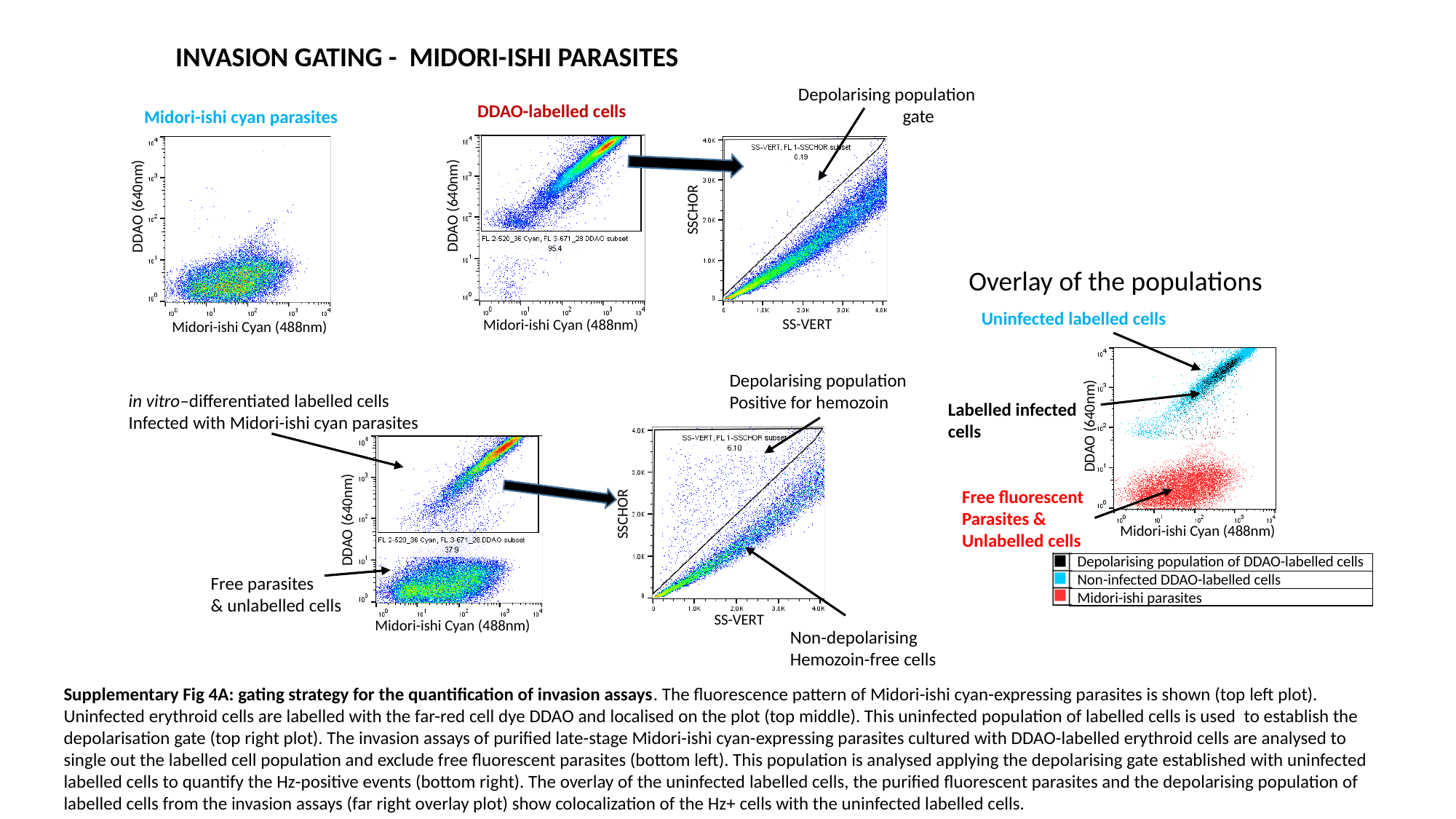

INVASION GATING - MIDORI-ISHI PARASITES
Depolarising population
 gate
SSCHOR
SS-VERT
DDAO-labelled cells
DDAO (640nm)
Midori-ishi Cyan (488nm)
Midori-ishi cyan parasites
DDAO (640nm)
Midori-ishi Cyan (488nm)
Overlay of the populations
Uninfected labelled cells
Labelled infected cells
DDAO (640nm)
Free fluorescent
Parasites &
Unlabelled cells
Midori-ishi Cyan (488nm)
Depolarising population of DDAO-labelled cells
Non-infected DDAO-labelled cells
Midori-ishi parasites
Depolarising population
Positive for hemozoin
SSCHOR
SS-VERT
Non-depolarising
Hemozoin-free cells
in vitro–differentiated labelled cells
Infected with Midori-ishi cyan parasites
DDAO (640nm)
Free parasites
& unlabelled cells
Midori-ishi Cyan (488nm)
Supplementary Fig 4A: gating strategy for the quantification of invasion assays. The fluorescence pattern of Midori-ishi cyan-expressing parasites is shown (top left plot). Uninfected erythroid cells are labelled with the far-red cell dye DDAO and localised on the plot (top middle). This uninfected population of labelled cells is used to establish the depolarisation gate (top right plot). The invasion assays of purified late-stage Midori-ishi cyan-expressing parasites cultured with DDAO-labelled erythroid cells are analysed to single out the labelled cell population and exclude free fluorescent parasites (bottom left). This population is analysed applying the depolarising gate established with uninfected labelled cells to quantify the Hz-positive events (bottom right). The overlay of the uninfected labelled cells, the purified fluorescent parasites and the depolarising population of labelled cells from the invasion assays (far right overlay plot) show colocalization of the Hz+ cells with the uninfected labelled cells.

## Slide 5
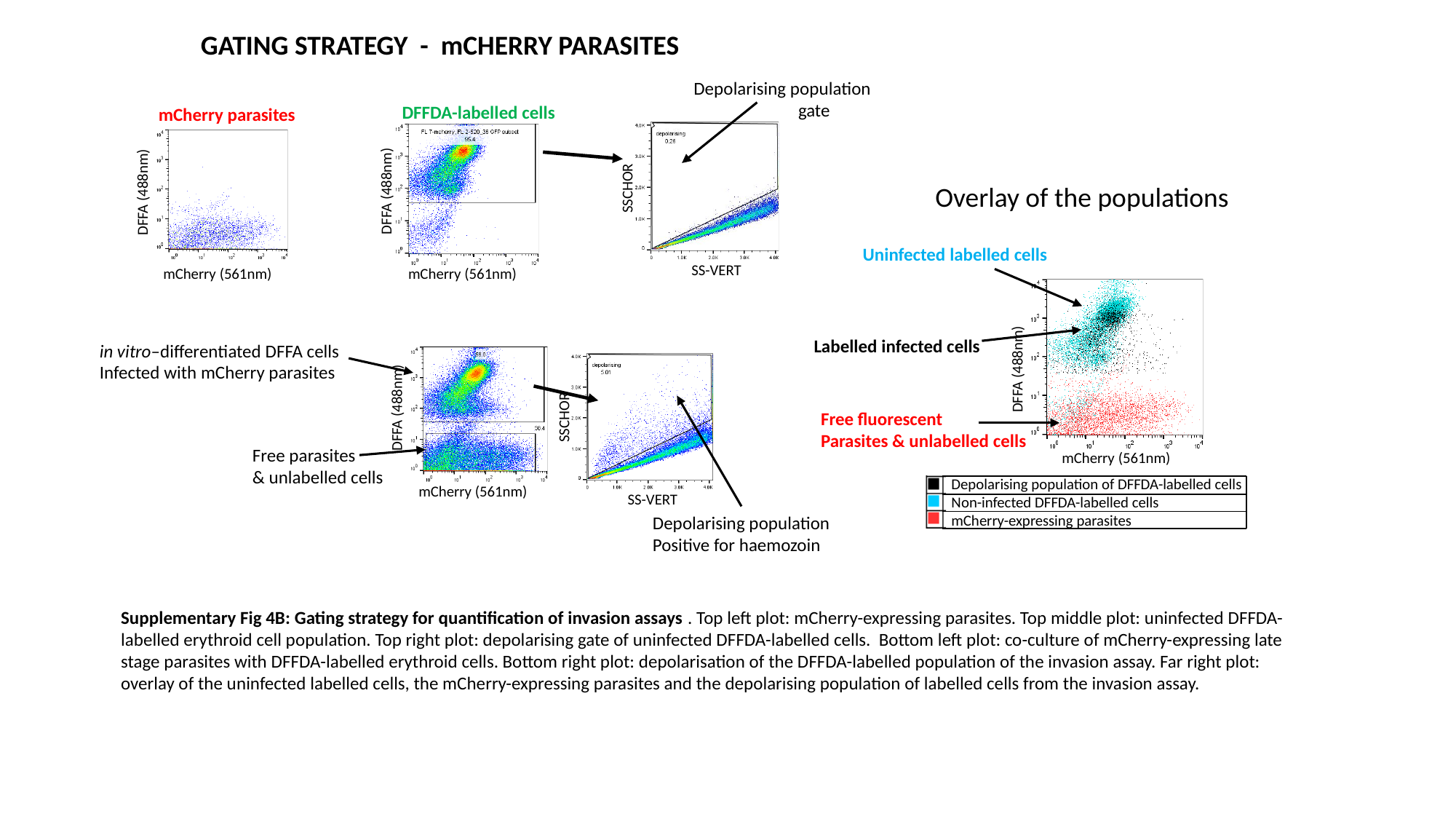

GATING STRATEGY - mCHERRY PARASITES
Depolarising population
 gate
SSCHOR
SS-VERT
DFFDA-labelled cells
DFFA (488nm)
mCherry (561nm)
mCherry parasites
DFFA (488nm)
mCherry (561nm)
Overlay of the populations
Uninfected labelled cells
Labelled infected cells
DFFA (488nm)
Free fluorescent
Parasites & unlabelled cells
mCherry (561nm)
Depolarising population of DFFDA-labelled cells
Non-infected DFFDA-labelled cells
mCherry-expressing parasites
in vitro–differentiated DFFA cells
Infected with mCherry parasites
DFFA (488nm)
SSCHOR
Free parasites
& unlabelled cells
mCherry (561nm)
SS-VERT
Depolarising population
Positive for haemozoin
Supplementary Fig 4B: Gating strategy for quantification of invasion assays . Top left plot: mCherry-expressing parasites. Top middle plot: uninfected DFFDA-labelled erythroid cell population. Top right plot: depolarising gate of uninfected DFFDA-labelled cells. Bottom left plot: co-culture of mCherry-expressing late stage parasites with DFFDA-labelled erythroid cells. Bottom right plot: depolarisation of the DFFDA-labelled population of the invasion assay. Far right plot: overlay of the uninfected labelled cells, the mCherry-expressing parasites and the depolarising population of labelled cells from the invasion assay.

## Slide 6
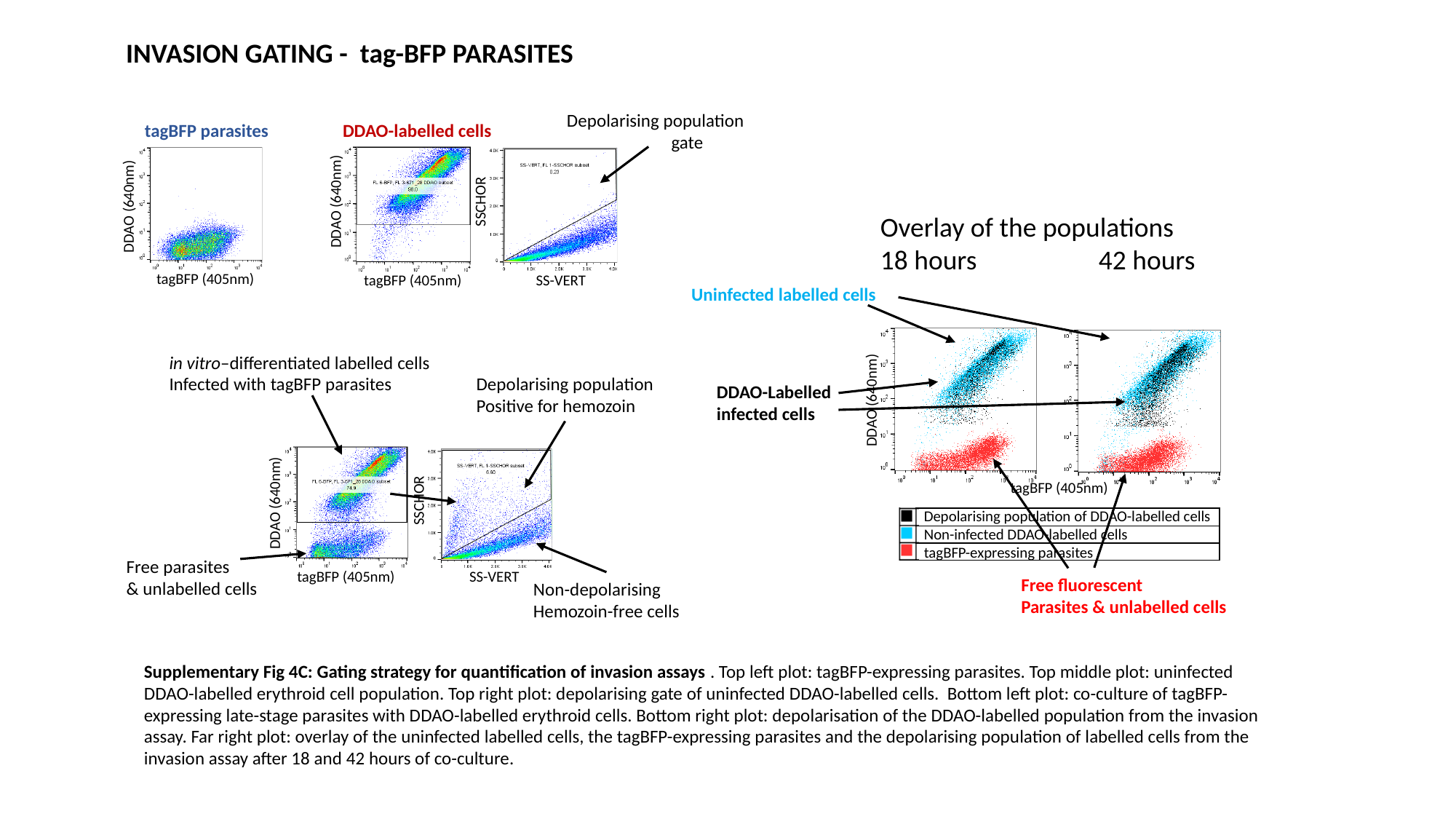

INVASION GATING - tag-BFP PARASITES
Depolarising population
 gate
DDAO-labelled cells
tagBFP parasites
SSCHOR
DDAO (640nm)
SS-VERT
tagBFP (405nm)
DDAO (640nm)
Overlay of the populations
18 hours		42 hours
tagBFP (405nm)
Uninfected labelled cells
in vitro–differentiated labelled cells
Infected with tagBFP parasites
Depolarising population
Positive for hemozoin
SSCHOR
DDAO (640nm)
Free parasites
& unlabelled cells
SS-VERT
tagBFP (405nm)
Non-depolarising
Hemozoin-free cells
DDAO-Labelled
infected cells
DDAO (640nm)
tagBFP (405nm)
Depolarising population of DDAO-labelled cells
Non-infected DDAO-labelled cells
tagBFP-expressing parasites
Free fluorescent
Parasites & unlabelled cells
Supplementary Fig 4C: Gating strategy for quantification of invasion assays . Top left plot: tagBFP-expressing parasites. Top middle plot: uninfected DDAO-labelled erythroid cell population. Top right plot: depolarising gate of uninfected DDAO-labelled cells. Bottom left plot: co-culture of tagBFP-expressing late-stage parasites with DDAO-labelled erythroid cells. Bottom right plot: depolarisation of the DDAO-labelled population from the invasion assay. Far right plot: overlay of the uninfected labelled cells, the tagBFP-expressing parasites and the depolarising population of labelled cells from the invasion assay after 18 and 42 hours of co-culture.

## Slide 7
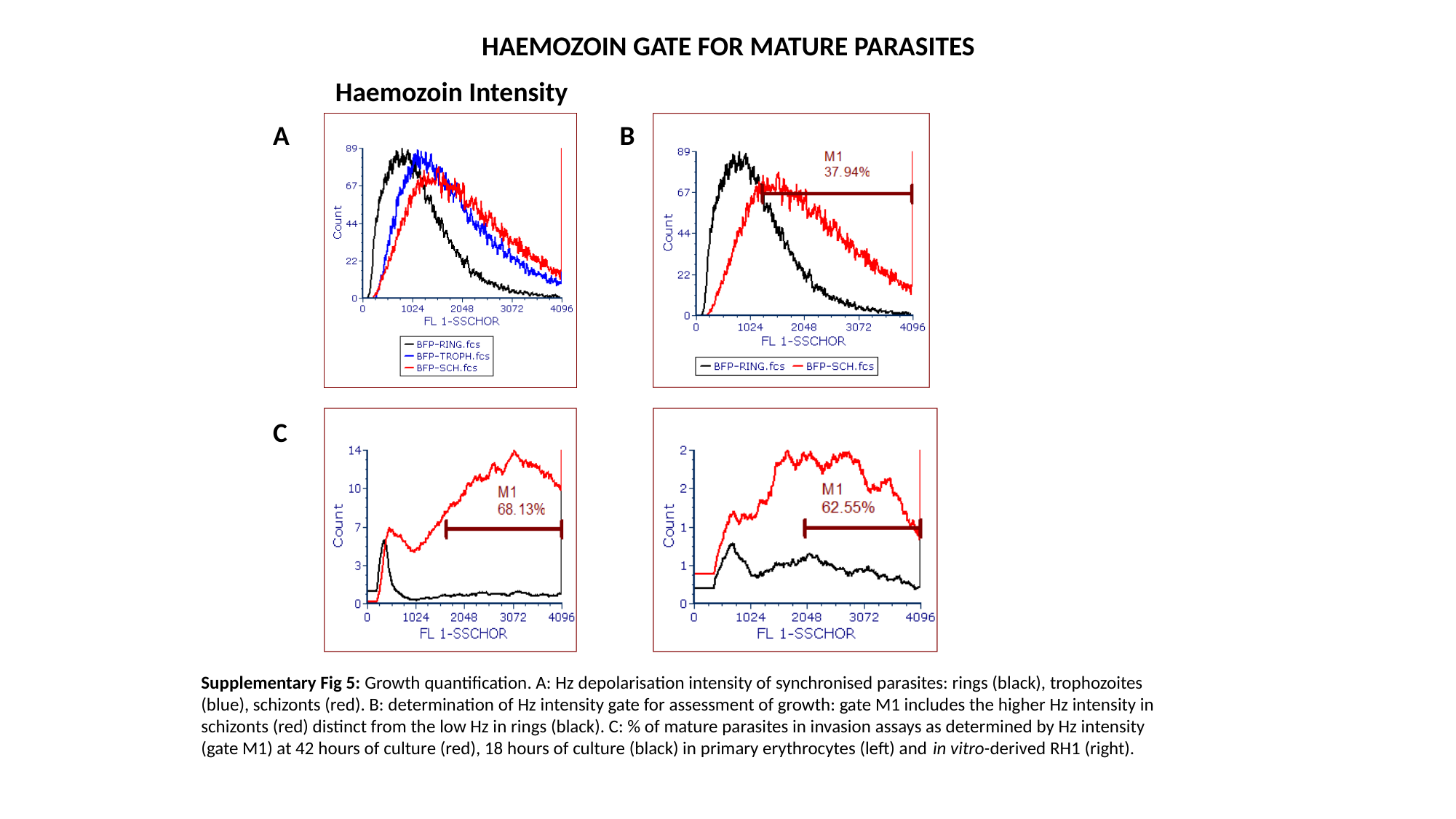

HAEMOZOIN GATE FOR MATURE PARASITES
Haemozoin Intensity
A
B
C
Supplementary Fig 5: Growth quantification. A: Hz depolarisation intensity of synchronised parasites: rings (black), trophozoites (blue), schizonts (red). B: determination of Hz intensity gate for assessment of growth: gate M1 includes the higher Hz intensity in schizonts (red) distinct from the low Hz in rings (black). C: % of mature parasites in invasion assays as determined by Hz intensity (gate M1) at 42 hours of culture (red), 18 hours of culture (black) in primary erythrocytes (left) and in vitro-derived RH1 (right).

## Slide 8
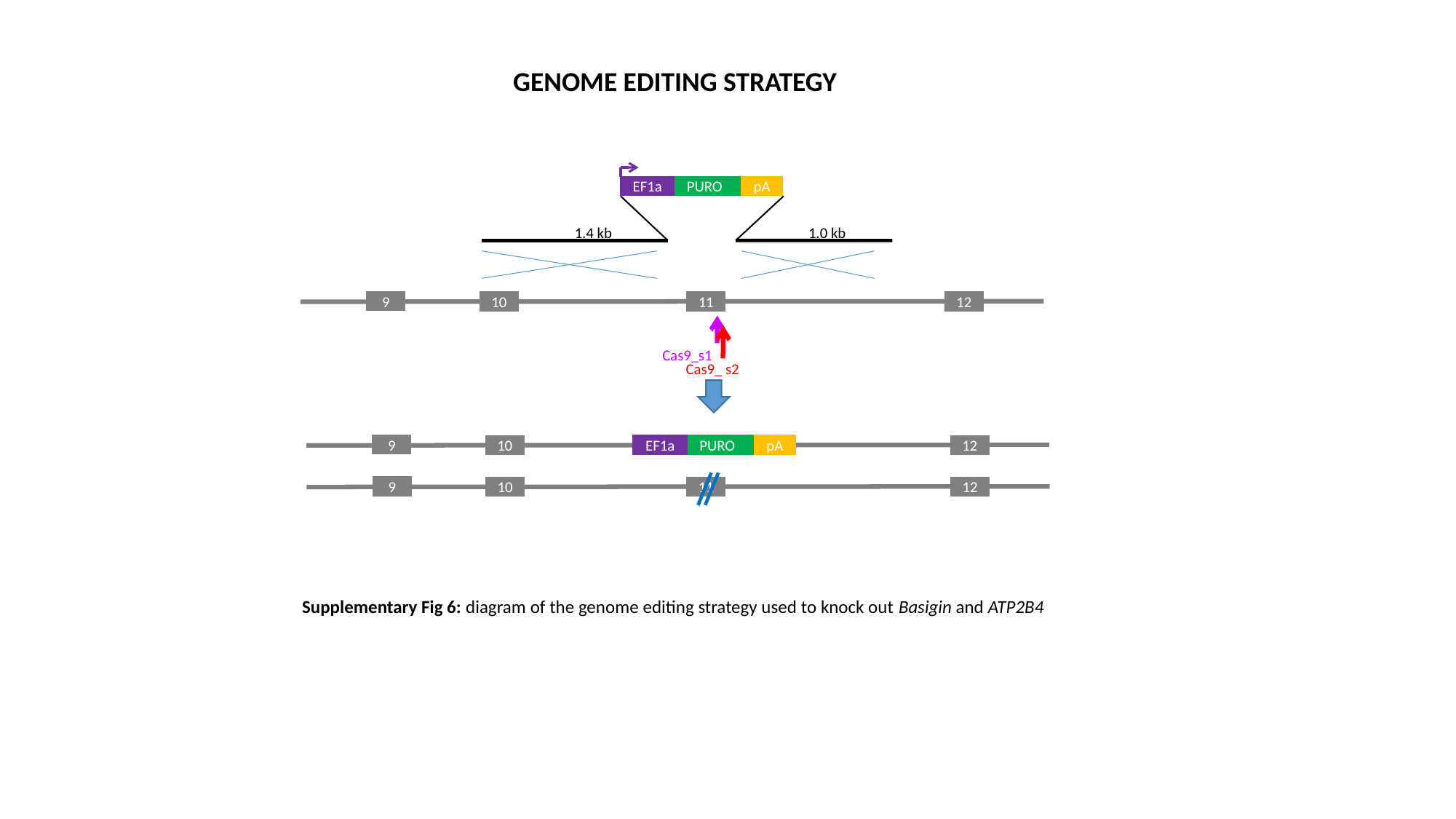

GENOME EDITING STRATEGY
EF1a
PURO
pA
1.4 kb
1.0 kb
9
10
11
12
Cas9_s1
Cas9_ s2
9
EF1a
PURO
pA
10
12
9
10
12
11
Supplementary Fig 6: diagram of the genome editing strategy used to knock out Basigin and ATP2B4

## Slide 9
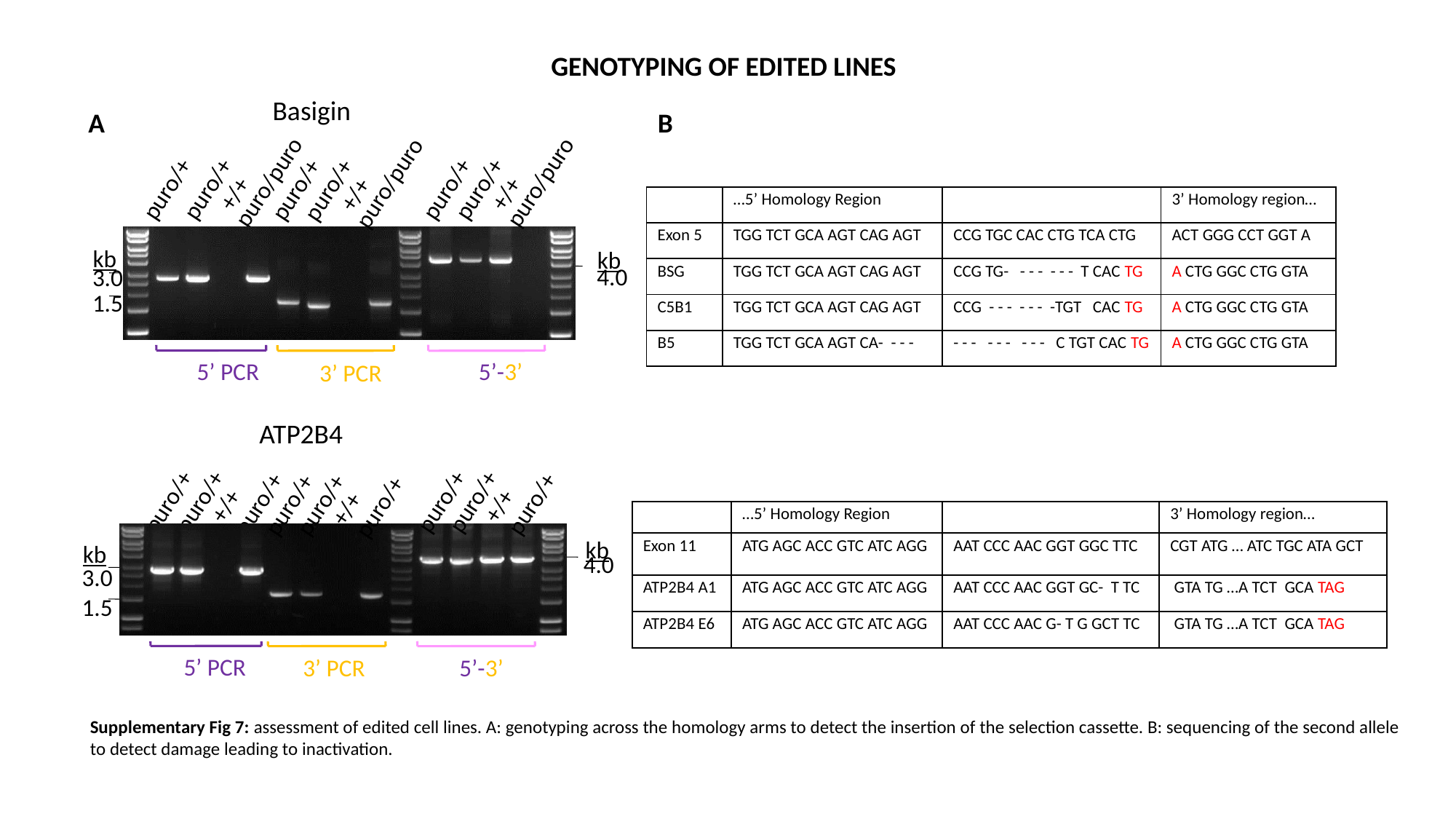

GENOTYPING OF EDITED LINES
Basigin
A
B
puro/puro
puro/+
+/+
puro/puro
puro/+
+/+
puro/puro
puro/+
+/+
puro/+
puro/+
puro/+
kb
kb
4.0
3.0
1.5
5’-3’
5’ PCR
3’ PCR
| | …5’ Homology Region | | 3’ Homology region… |
| --- | --- | --- | --- |
| Exon 5 | TGG TCT GCA AGT CAG AGT | CCG TGC CAC CTG TCA CTG | ACT GGG CCT GGT A |
| BSG | TGG TCT GCA AGT CAG AGT | CCG TG- - - - - - - T CAC TG | A CTG GGC CTG GTA |
| C5B1 | TGG TCT GCA AGT CAG AGT | CCG - - - - - - -TGT CAC TG | A CTG GGC CTG GTA |
| B5 | TGG TCT GCA AGT CA- - - - | - - - - - - - - - C TGT CAC TG | A CTG GGC CTG GTA |
ATP2B4
puro/+
+/+
puro/+
+/+
puro/+
+/+
puro/+
puro/+
puro/+
puro/+
puro/+
puro/+
kb
kb
4.0
3.0
1.5
5’ PCR
5’-3’
3’ PCR
| | …5’ Homology Region | | 3’ Homology region… |
| --- | --- | --- | --- |
| Exon 11 | ATG AGC ACC GTC ATC AGG | AAT CCC AAC GGT GGC TTC | CGT ATG … ATC TGC ATA GCT |
| ATP2B4 A1 | ATG AGC ACC GTC ATC AGG | AAT CCC AAC GGT GC- T TC | GTA TG …A TCT GCA TAG |
| ATP2B4 E6 | ATG AGC ACC GTC ATC AGG | AAT CCC AAC G- T G GCT TC | GTA TG …A TCT GCA TAG |
Supplementary Fig 7: assessment of edited cell lines. A: genotyping across the homology arms to detect the insertion of the selection cassette. B: sequencing of the second allele to detect damage leading to inactivation.
